# Comparison of the transcriptomes of American chestnut (*Castanea dentata*) and Chinese chestnut (*Castanea mollissima*) in response to the chestnut blight infection

**DOI:** 10.1186/1471-2229-9-51

**Published:** 2009-05-09

**Authors:** Abdelali Barakat, Denis S DiLoreto, Yi Zhang, Chris Smith, Kathleen Baier, William A Powell, Nicholas Wheeler, Ron Sederoff, John E Carlson

**Affiliations:** 1 The School of Forest Resources, Department of Horticulture, The Huck Institutes of the Life Sciences, The Pennsylvania State University, 323 Forest Resources Building, University Park, PA 16802, USA; 2 Forest Biotechnology Group, North Carolina State University, Raleigh, North Carolina 27695, USA; 3 Department of Environmental Science and Forestry, State University of New York, Syracuse, NY, USA

## Abstract

**Background1471-2229-9-51:**

American chestnut (*Castanea dentata*) was devastated by an exotic pathogen in the beginning of the twentieth century. This chestnut blight is caused by *Cryphonectria parasitica*, a fungus that infects stem tissues and kills the trees by girdling them. Because of the great economic and ecological value of this species, significant efforts have been made over the century to combat this disease, but it wasn't until recently that a focused genomics approach was initiated. Prior to the Genomic Tool Development for the Fagaceae project, genomic resources available in public databases for this species were limited to a few hundred ESTs. To identify genes involved in resistance to *C. parasitica*, we have sequenced the transcriptome from fungal infected and healthy stem tissues collected from blight-sensitive American chestnut and blight-resistant Chinese chestnut (*Castanea mollissima*) trees using ultra high throughput pyrosequencing.

**Results:**

We produced over a million 454 reads, totaling over 250 million bp, from which we generated 40,039 and 28,890 unigenes in total from *C. mollissima *and *C. dentata *respectively.

The functions of the unigenes, from GO annotation, cover a diverse set of molecular functions and biological processes, among which we identified a large number of genes associated with resistance to stresses and response to biotic stimuli. *In silico *expression analyses showed that many of the stress response unigenes were expressed more in canker tissues versus healthy stem tissues in both American and Chinese chestnut. Comparative analysis also identified genes belonging to different pathways of plant defense against biotic stresses that are differentially expressed in either American or Chinese chestnut canker tissues.

**Conclusion:**

Our study resulted in the identification of a large set of cDNA unigenes from American chestnut and Chinese chestnut. The ESTs and unigenes from this study constitute an important resource to the scientific community interested in the discovery of genes involved in various biological processes in Chestnut and other species. The identification of many defense-related genes differentially expressed in canker vs. healthy stem in chestnuts provides many new candidate genes for developing resistance to the chestnut blight and for studying pathways involved in responses of trees to necrotrophic pathogens. We also identified several candidate genes that may underline the difference in resistance to *Cryphonectria parasitica *between American chestnut and Chinese chestnut.

## Background

The chestnuts (*Castanea*), members of the family Fagaceae, naturally occur throughout deciduous forests of eastern North America, Europe, and Asia [1]. The genus includes ecologically and economically important nut and timber producing trees including the Chinese chestnut (*Castanea mollissima*), Japanese chestnut (*Castanea crenata)*, European Chestnut (*Castanea sativa) *and American chestnut (*Castanea dentata*).

American chestnut was once a dominant tree species in forest ecosystems of eastern North America, its range extending from Maine south along the Appalachian Mountains to Alabama and westward to the Mississippi river [2]. In some areas up to 45% of the forest canopy was comprised of American chestnut [3]. This large, fast-growing tree played a central role in forest ecosystems, providing food and habitat for a variety of wildlife. It was also of considerable economic importance, producing strong, rot-resistant timber, a source of tannins, fuel, wood, and nuts [4-6]. Because of its utility, rapid growth, ability to quickly colonize burned or clearcut areas, and edible nuts it has been referred to as the "perfect tree" [5].

The reign of the American chestnut came to an abrupt end in the early 1900's when a blight, caused by the fungus, *Cryphonectria parasitica*, was introduced to North America from Asia via infected chestnut nursery stock [2]. The blight was first observed in the Bronx Zoological Park in New York in 1904 [7] and within 50 years the American chestnut was nearly eliminated from the forest [8]. The pathogen infects stem tissues and kills the above ground portions of trees by girdling them. Below ground the trees can survive for many years however, continuously sending up sprouts which are themselves eventually infected. *Cryphonectria*, which shows a necrotrophic life style is lesser studied than their biotrophic counterparts. Today, except for occasional trees near the edge of its range which have escaped the blight, American chestnut exists primarily as shrubs, sprouting from the stumps of blight-topped trees [2,9].

Although to a lesser extent, European chestnut (*C. sativa) *was also devastated by introduction of *C. parasitica *[10]. Despite their close relationship, sister species of *Castanea *exhibit very different susceptibilities to *Cryphonectria *infection. Asian chestnuts, the vector for the spread of *Cryphonectria *westward, range from somewhat susceptible to nearly immune to infection [4]. Most likely, these species co-evolved with *Cryphonectria*. Slow growing cankers are often visible on Chinese and Japanese chestnut trees although growth and yield of the trees are not substantially reduced. European chestnut is able to tolerate infection slightly more than American chestnut, which has little or no natural resistance to *Cryphonectria *infection [7].

Multiple attempts are being made to develop blight-resistant American chestnut genotypes. The search for natural resistance within American chestnut has been mostly fruitless whereas crosses between American parents exhibiting limited resistance have produced progeny without appreciable resistance [2]. The American Chestnut Foundation [11] has been breeding for resistance for over three decades by introgression of genes from Chinese chestnut into American chestnut. However, this approach, although successful in developing blight resistant American chestnut varieties, has been slowed by the lack of genetic tools. Another approach to restoration of chestnut is by introduction of hypovirulent genotypes of the pathogen, *Cryphonectria parasitica *[10]. Hypovirulence is a process in which the virulence of *C. parasitica *to chestnut trees is reduced by its infection by fungal viruses. For instance, virus-infected individuals of C. parasitica have been shown to produce superficial non-lethal cankers on European chestnut, and regular treatments with the virus are employed to protect chestnut farms in Europe. However, attempts to inoculate existing American Chestnut cankers with hypovirulent strains have met with limited success and may be impractical for reducing blight symptoms in the forest due to the large scale of the land mass affected [2].

Development of genomic tools will certainly facilitate the isolation of resistance genes, improve the efficiency of backcross breeding, and provide genetic reagents for developing resistant varieties by genetic engineering. American Chestnut is transformable using *Agrobacterium tumefaciens *[12,13] and methods for plant regeneration from somatic embryos have been developed [14-16], permitting the production of many individuals from single transformation events. C.A. Maynard's and W.A. Powell's labs have produced transgenic American chestnut trees that are in their second year of field trials (USDA APHIS BRS permit 08-011-105r) demonstrating that all the steps have been developed to genetically engineer this species.

Genomic tools are now being developed to accelerate the identification of resistance genes and the development of blight resistant American chestnut. In this context, a central objective of The Fagaceae Genomic Tools Project [17] is the sequencing of the transcriptomes of chestnut, oak and beech species with the long-term goal of isolating genes underlying resistance to the chestnut blight. In this study we used an ultra-high throughput pyrosequencing approach [18] to quickly generate millions of bases of cDNA sequence for plant transcriptome analysis [19-22]. A comparison of capillary sequencing and next generation sequencing methods [23] showed that pyrosequencing is well adapted for analyzing the transcriptome of both model and non-model species, with lower cost than conventional methods such as microarrays, SAGE, or EST analysis generated using capillary sequencing.

In total, for all tissues, we have generated and analyzed 317,842 and 856,618 sequence reads from American and Chinese chestnut, respectively, for which the Fasta files can be accessed at the Fagaceae project website [17] and the raw data files in the Short Read Archive at the National Center for Biotechnology Information [24], accession numbers SRX001799 to SRX001808. Here we focused on comparing the transcriptomes generated from healthy stems and infected canker tissues from American and Chinese chestnut. The comparison between the American and Chinese chestnut canker transcriptomes enabled us to identify a large number of candidate pathogen response genes for use in studying pathways involved in resistance to the chestnut blight.

## Results

### 454 sequence from Canker tissue libraries

The American Chestnut Canker cDNA library was constructed from a pool of RNA isolated from canker tissues of several individuals of one genotype (BA69). The Chinese chestnut cDNA library was also prepared from RNA extracted from several individuals of a single genotype (Nanking). One plate of sequencing was conducted with each library using the GS20 model of the 454 system. A total of 129,508 and 235,635 reads were generated from American and Chinese chestnut canker transcriptomes respectively, in the GS20 runs (NCBI SRA accessions SRX001804 and SRX001799, respectively). The average length of the reads was 101 nucleotides (nt) (Table 1). The difference in the number of reads generated for the American and the Chinese chestnut canker reflects the lower quality of the American chestnut library. In total ~13.3 and ~24.0 megabases of cDNA were generated from the American and Chinese chestnut canker libraries, respectively. Prior to assembly, the canker raw sequence data from the GS20 was re-analyzed with the improved base-calling software of the new FLX model 454 sequencer. Contig construction of the 454 reads using the Newbler assembly software (454 Life Sciences) led to the construction of 7,171 and 14,308 contigs from American and Chinese chestnut, respectively (Table 1). Among those contigs, 247 and 436 were considered large, having an average length of 731 nt. There were also 68,860 and 100,901 sequences from American and Chinese chestnut cankers, respectively, that did not overlap with other sequences and were considered as singletons. From the canker transcript contigs we were able to tag 5,636 genes from American chestnut and 8,369 from Chinese chestnut. When those unigenes (transcript contigs) were queried using BlastX (e-value cutoff: e-10) against the *Cryphonectria parasitica *proteome, significant matches were found for 102 (~1.6%) and 213 (~1.5%) of the American and Chinese chestnut unigenes, respectively.

**Table 1:** Summary of 454 sequencing results obtained in this study for the American and Chinese chestnut transcriptomes

**Sample**	**System**	**# Plates**	**# of Reads**	**# of bp**	**AL of Reads**	**# Contigs**	**AL of All Contigs**	**# Large Contigs**	**AL of Large Contigs**
ACCanker	GS20	1	129,508	13,080,308	101	7,171	168	247	689
ACHS1	FLX	3/4	126,791	29,828,910	247	11,496	276	885	817
ACHS2	FLX	3/4	162,624	38,165,054	246	9,431	271	691	816
CCCanker	GS20	1	235,635	23,799,135	101	14,308	168	436	773
CCMHS	FLX	3/4	228,594	56,051,191	246	21,828	344	3,074	851
CCNHS	FLX	3/4	259,859	64,271,926	247	28,784	339	4,451	848
Total	both	5	2,184,941	428,799,020	198	93.018	261	9,784	799

#, Number; AL, Average length

ACCanker, American chestnut genotype BA69 infected stem (canker) cDNA

CCCanker, Chinese chestnut variety 'Nanking' infected stem (canker) cDNA

CCMHS, Chinese Chestnut variety 'Mahogany' healthy stem cDNA

CCNHS, Chinese Chestnut variety 'Nanking' healthy stem cDNA

ACHS1, American Chestnut Wisneiwski genotype healthy stem cDNA

ACHS2, American Chestnut Watertown genotype healthy stem cDNA

### 454 sequence from healthy stem tissue libraries

Four libraries were constructed from American and Chinese chestnut healthy stem tissues. For Chinese chestnut, two separate libraries were constructed from healthy cambial tissue collected from blight resistant genotypes 'Nanking' and 'Mahogany'. For American chestnut two libraries were constructed from the genotypes Watertown and Wisniewski. In contrast to the canker transcriptome sequencing, American chestnut and Chinese chestnut healthy stem transcriptomes were sequenced using the FLX system (Roche). A quarter plate of sequencing was conducted for each American chestnut healthy stem library, while a three quarter plate worth of sequencing were conducted for the Chinese chestnut Nanking and Mahogany libraries (Table 1). Sequencing of the healthy stem transcriptome from the two American chestnut genotypes yielded a total of 188,334 reads (NCBI SRA accessions SRX001800 and SRX001801, respectively), with an average read length of ~246 nt (Table 1). Slightly more than 2.5 times that number of reads (488,453) was generated from the two Chinese chestnut healthy stem genotypes (NCBI SRA accessions SRX001805 and SRX001806, respectively) with an average read length of 247.9 bases (Table 1). In total, ~46.4 and ~120 megabases of healthy stem transcriptome sequence were obtained from American and Chinese chestnut healthy stems, respectively. We generated 20,927 contigs for American chestnut healthy stem and 50,612 contigs for Chinese chestnut healthy stem using the Newbler assembly software package (454 Life Sciences) with an average length of 273 nt and 330 nt, respectively. A total of 1,823 and 7,961 contigs from American and Chinese chestnut, respectively, were considered large with an average length of 833 nt. This left 95,483 and 100,779 unassembled singleton reads from American and Chinese chestnut healthy stem sequences, respectively. From the contigs, a total of 12,883 and 15,085 genes were tagged from American chestnut and Chinese chestnut healthy stem tissues, respectively.

### Functional annotation of American and Chinese chestnut

**Figure 1:**
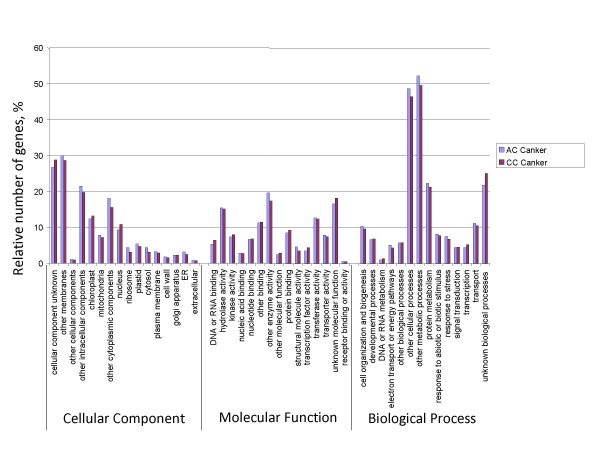
**Histogram presentation of Gene Ontology classification of putative molecular function of unigenes from American and Chinese chestnut canker tissues and biological processes in which they are involved**.

**Figure 2:**
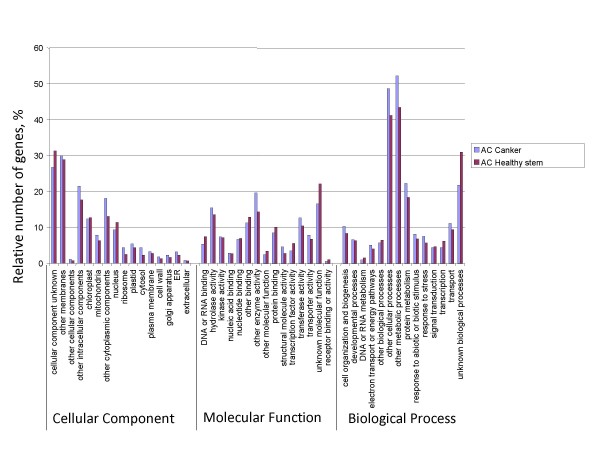
**Histogram presentation of Gene Ontology classification of putative molecular functions of unigenes from American chestnut healthy stem and canker tissues and biological processes in which they are involved**.

**Figure 3:**
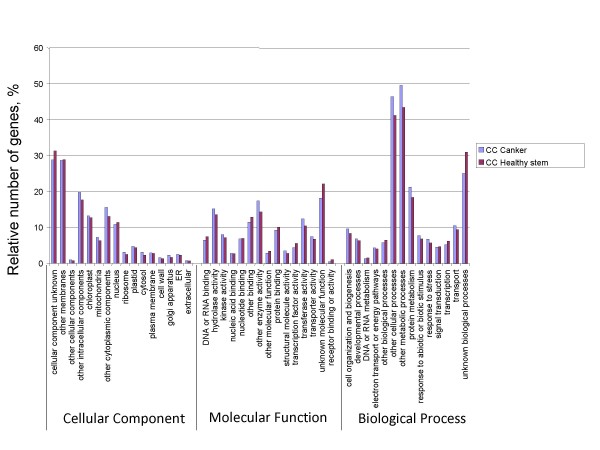
**Histogram presentation of Gene Ontology classification of putative molecular functions of unigenes from Chinese chestnut healthy stem and canker tissues and biological processes in which they are involved**.

To determine the possible functions of genes tagged, we used the Gene Ontology (GO) [25] classification system. Based on the *Arabidopsis *proteome, a function could be assigned to 83,292 (26%) and 20,391 (28%) of the 454 reads from American and Chinese chestnut respectively. These percentages are lower than those obtained by BLASTx alignments to the *Populus *proteome (39% and 46% for American and Chinese chestnut respectively). However, most of the reads (45,804 and 139,230 for American and Chinese chestnut respectively) with best hits to the *Populus *proteome are for *Populus *genes that are annotated as having no known function. GO ontology analysis based on the *Arabidopsis *proteome showed that the distributions of gene functions for cDNA sequences from American and Chinese chestnut cankers are similar (Fig. 1). This expected result indicates that there is no bias in the construction of the libraries from American and Chinese canker tissues. The functions of genes identified cover various biological processes. However, hydrolase and transferase are among the most represented molecular function categories. The biological processes most represented were transport and protein metabolism. It is noteworthy that a larger number of genes involved in response to biotic and abiotic stimuli and stresses were identified in Chinese chestnut tissues compared with American chestnut. This difference may be associated with blight resistance in Chinese chestnut. A similar pattern of GO-annotation function distribution was found when the transcriptomes from healthy stem and canker tissues from American and Chinese chestnut were compared (Fig. 2 and Fig. 3). As predicted, we observed that the fraction of genes involved in response to stress, biotic and abiotic stimuli, cell organization and biogenesis processes are highly represented. The molecular functions most represented are transferase, protein binding, and hydrolase.

### Transcriptome comparison between canker and healthy stem tissues within Chinese and American chestnut

To determine the effect of the infection by the blight causing fungus on gene expression in American and Chinese chestnut trees, we compared the transcriptomes from cankered versus healthy stems (Fig. 2 and Fig. 3). We first determined how many times a unigene was represented in each of the libraries based on the number of reads for each (unigene count). We then determined which genes were in common in the two transcriptomes, versus being specific to a library, based on searching the GenBank Accession numbers of the contigs and reads as annotated by BlastX alignment to the Arabidopsis proteome. Analysis of canker and healthy stem transcriptomes from American and Chinese chestnut showed that several resistance-related genes were differentially expressed in canker tissues (see Additional file 1 and Additional file 2). Those genes encode various transcription factors such as WRKY, zinc finger, Myb, C2 domain, basic helix-loop-helix, CCAAT-box, and CCR4-NOT. Several other genes involved in resistance to biotic stresses were differentially expressed in canker tissues. Those genes include *cinnamoyl-CoA reductase *(*CCR*), *4-coumarate–CoA ligase*, *hydrolase*, *kinases*, *phosphatases*, *translation factor, ATPases*, *pathogen responsive alpha-dioxygenase*, etc (see Additional file 3 and Additional file 4). Some genes such as *ABC transporter, CCAAAT-box, CCR4-NOT *and *zinc finger *seem to be differentially expressed in canker *vs*. healthy stem tissues in Chinese chestnut.

### Transcriptome comparison between Chinese and American chestnut canker tissues

To gain insight into the differences in the response of the American and Chinese chestnut species to infection by the blight-causing agent, we compared the transcriptomes from Chinese chestnut canker tissues and American chestnut canker tissues (Fig. 1) as described above. This comparison showed that the distribution of gene functions was very similar overall in both the cankers of both species. However, we observed a small increase in nucleic acid protein binding and transcription factor molecular functions in Chinese chestnut cankers. In an opposite pattern, American chestnut had a small increase in the category "structural molecule activity". We also observed that the fraction of genes involved in response to stress and biotic and abiotic stimuli was slightly higher in American chestnut canker tissue. However, statistical analysis using GOstat program showed that none of those differences were statistically significant [26]. Detailed comparison of the transcriptomes showed that many resistance-related genes were differentially expressed in both the American and the Chinese chestnut infection sites (see Additional file 3 and Additional file 4). Statistical tests as per [27] of the expression data showed that the differential expression of many of the resistance-related genes was statistically significant. Examples of the resistance-related genes preferentially expressed in American chestnut (based on the difference in reads per unigene) include genes encoding proteins such as SNF7, laccase, CCR, cinnamyl alcohol dehydrogenase (CAD), expansin, F-box proteins, FAD-binding protein, proteins named disease-resistance-responsive, etc. Most of those genes play an important role in plant response to pathogen infection. Genes presenting relatively high expression in Chinese chestnut encode proteins such as mitogen-activated kinase, Myb transcription factors, pathogen-responsive alpha-dioxygenase, laccase, cytochrome P450, F-box proteins, SNF7, CCR, succinyl-CoA ligase, etc. However, most of the gene expression differences in Chinese chestnut were not statistically significant in our data set.

## Discussion

### American and Chinese chestnut transcriptome sequencing

Advances in DNA sequencing technology during the last decade have dramatically impacted genome sequencing and transcriptome analysis. Techniques such as microarrays and SAGE have facilitated transcriptome analysis at large scale from numerous plants. However, those techniques could be used only for model plants with known genome sequences. EST sequencing has been successfully used to analyze the transcriptome in non model plants. However, deep EST sequencing using capillary sequencing, which requires cDNA cloning and individual DNA preparations for each clone, is time consuming and very costly. Bead-based pyrosequencing introduced recently [18] constitutes a better alternative for transcriptomics. The high number of reads generated per run together with the low sequencing error rate in the contigs obtained makes it a good tool to deeply sequence the transcriptome of plants. This approach has been used successfully for analyzing the transcriptomes of maize and *Arabidopsis *[19-22] and we have applied it to the non-model tree species *Castanea dentata *and C. *mollissima*.

Before this project, only a few hundred chestnut sequences had been deposited in the EST database (dbEST) at NCBI. The data presented here represent the first large effort by the Fagaceae Genomic Tools Development project to generate cDNA resources and analyze the transcriptomes of American and Chinese chestnut. These resources are public and the sequences can be accessed in a searchable database at the project website [17], or as raw sequence data at the NCBI Short Read Archive (accession above). In total, our study generated 171 Mb and 78 Mb and tagged 40,039 and 28,890 genes from Chinese chestnut and American chestnut, respectively. A fraction (ranging between 14% and 21%) of American and Chinese chestnut unigenes that could not be annotated using the *Arabidopsis *proteome could however be annotated using the *Populus *proteome. Most of the genes with no hits to the *Arabidopsis *proteome encoded proteins annotated with unknown functions in the poplar genome, however. Those genes could correspond to either tree specific genes or sequences that have diverged in *Populus *and chestnut beyond recognizable homology to *Arabidopsis *using the Blast algorithm. Moreover, over 50% of the 454 reads could not be annotated using either the *Arabidopsis *proteome or the *Populus *proteome. A query against the Fungi database at NCBI excluded a *Cryphonectria *origin for a small fraction of those reads (~3% for both species). While a fraction of the remaining sequences may correspond to 3' or 5' untranslated regions, non coding RNAs, or short sequences not containing a known protein domain, a large number may correspond to potential Chestnut-specific genes. A similar situation was found when analyzing the transcription of *Eschscholzia californica*, *Persea americana*, and *Aristolochia fimbriata *[28] and (Kerr Wall, personal communication). The two sets of unigenes from Chinese chestnut and American chestnut also include a large number of genes known to be involved in response to biotic and abiotic stimuli and stress in general. These gene sequences constitute a very important resource to the scientific community working on chestnut blight resistance as well as those interested in gene discovery in Fagaceae species.

By taking into consideration only the sequences that have homologies in the *Arabidopsis *proteome, two plates of 454 sequences from American chestnut and Chinese chestnut were enough to generate ~13,000 and ~15,000 unigenes from each species. This number represents 52% and 60% of American and Chinese chestnut transcriptome respectively, assuming that the two chestnut species have a similar gene number as *Arabidopsis*. Such breadth and depth (the number of reads per gene varying between 60 and 178) of coverage by 454 gene tagging, makes this technique a good tool for quantifying the expression level of sets of genes involved in various developmental stages or physiological conditions. cDNA sequences generated from both species cover various biological processes and molecular functions indicating that 454 sequencing constitutes a powerful tool for sequencing the transcriptome of non model species. These results confirm that pyrosequencing constitutes a powerful tool for transcriptome characterization and gene discovery.

### Transcriptome comparison between canker tissues from *Castanea mollissima *and *Castanea dentata*

GO annotation analyses showed that, overall, canker tissues from both species present a similar transcriptome. Gene function categories associated with metabolic process are highly represented in both transcriptomes. The category represented the most is composed of genes associated with various metabolic processes as previously described in other systems such as cassava [29]. The second most highly represented category includes genes involved in resistance to stress and response to biotic and abiotic stimuli. Detailed analysis of the 454 sequences from both Chinese and American chestnut showed that the tagged genes included a large number associated with resistance to biotic and abiotic stresses. These include genes involved in pathogen recognition and signaling, transcription factors, and resistance genes. Comparison of genes highly expressed in the canker tissues of both American and Chinese chestnut showed that a fraction were either preferentially expressed in American chestnut or in Chinese chestnut. Genes with hydrolase activity represented the functional category with the largest number of members (unigenes or reads). Two members of the hydrolase group are the glycosyl hydrolase family 3 proteins, each of which was found five times in our transcriptome data. Glycosyl hydrolases break the bonds between carbohydrates and are involved in expansion and degradation of cell walls [30]. It is possible that some of the genes with hydrolase activity identified in Chinese chestnut canker tissue are contaminants from the pathogen fungi (*Cryphonectria*) mycelium within the canker and function by weakening the plant cell wall to facilitate fungal entry. However, analysis of these hydrolase sequences showed that they are more similar to other plant hydrolase genes than to fungal hydrolase sequences. This suggests that these hydrolase proteins are of plant origin and function either by strengthening cell walls against pathogen entry or in the programmed cell death response of the cells at the fungal infection site in the chestnut stem. A second functional category well represented is kinase activity. Such genes are involved in signaling in pathogen infection and play a key role in plant defense response. A third functional category observed in the chestnut transcriptomes is represented by transcription factors or genes associated with RNA or protein binding. Such genes may modulate the expression of resistance genes in response to the pathogen infection.

### Candidate genes involved in chestnut response to *Cryphonectria parasitica *infection

Among genes that were found to be differentially expressed in American or Chinese chestnut or both, several are known to be involved in various processes of plant defense against pathogens such as cell death related to hypersensitivity response, construction of a physical barrier to block the pathogen progression, as well as systemic resistance. Among genes involved in hypersensitivity cell death, we found *ABC transporter*, *C2-domain-containing gene*, *methylenetetrahydrofolate reductase*, *elongation factor-1 alpha*, and *peroxidase*. Such genes are involved in controlling the extent of the cell death in the defense response [31-34]. Pleiotropic drug resistance genes (ABC transporter family), which are involved in jasmonic acid pathway response, induce the secretion of secondary metabolites such as diterpenes that inhibit the growth of invading organisms [35-37]. The other category of genes that seems to be involved in plant resistance to the pathogen encodes proteins involved in lignin biosynthesis such as CCR, CAD, o-methyltransferase 1, cytochrome P450, 4-coumarate–CoA ligase, succinyl-CoA ligase, S-adenosylmethionine synthase 3, and S-adenosylmethionine synthase 2. Previous studies [38-42] showed that genes involved in lignin synthesis are over-expressed in various plants when they were challenged with pathogens. Among other resistance genes over-expressed in American and Chinese chestnut, we found several laccase genes, which also belong to the phenylpropanoid pathway. Polyphenol oxidases (PPO) catalyzing the oxygen-dependent oxidation of phenols to quinines, have been demonstrated to increase tomato plant resistance against *Pseudomonas syringae *[43]. We also found several ATP-binding cassette transport proteins, which are involved in both constitutive and jasmonic acid-dependent induced defense [35]. Chestnut plants seem also to activate the expression of genes involved in systemic resistance when they are challenged with the blight fungus. Among genes belonging to this pathway, we identified *omega-3 fatty acid desaturase*, *suppressor of fatty acid desaturase deficiency *(*SFD1 *and *SFD2*), *Ras-related GTP-binding *which are required for systemic resistance [44,45]. *ATPase *was found to be over-expressed in American and Chinese chestnut. This gene is required for the attenuation of the hypersensitive response [43]. Among genes involved in signaling, we found several genes such as *mitogen activated protein*. This protein kinase activates both local resistance and basal resistance [38,46,47]. It also appears from our data that metabolic flux may be involved in the chestnut resistance to the fungus. Several other genes involved in the regulation of resistance gene expression such as *Acetyl co-enzyme A carboxyltransferase *(*CAC3*), *SNF*, and several transcription factors such as *WRKY*, *Zinc finger*, *Myb*, etc were identified. *Myb *genes are involved in regulation of disease resistance genes [48-50]; they regulate the expression of the gene *PAL2*, a key enzyme in phenylpropanoid and lignin biosynthesis [51]. *WRKY *transcription factors have been shown to fine tune the response of plants to challenge with pathogens [52]. *SNF *genes interact with other genes, such as *SnRK1*, which regulate glucose metabolism, cell defense and other cellular processes.

Overall, this study allowed us to conclude that chestnut trees respond to *Cryphonectria parasitica *infection by activating both local and systemic resistance responses. The trees try first to block the progression of the pathogen by increasing the expression of hydrolases, lignin synthesis, and cell death. The infection is also sensed by *mitogen kinases*, which activate other transcriptions factors such as *AP2*, *Myb*, and *WRKY*, which in turn induce the expression of genes from the phenylpropanoid, jasmonic acid, oligochitosan, and other pathways that are involved in resistance to pathogens [53,54].

## Conclusion

In conclusion, this study allowed us to (i) Obtain over 28,000 and 40,000 unigenes from American and Chinese chestnut, (ii) Compare the transcriptomes of American and Chinese chestnut following infection by *Cryphonectria parasitica*, (iii) Identify potential pathways involved in chestnut resistance to the *Cryphonectria parasitica*, and (iv) Identify several candidate genes for resistance to necrotrophic fungal pathogens in trees.

## Methods

### American and Chinese chestnut materials

Healthy cambial tissue was collected from the American chestnut genotypes 'Watertown' and "Wisniewski' growing at the Connecticut Agricultural Experiment Station, Lockwood Farm, Hamden CT. Canker tissue was collected from the American chestnut genotype BA69 growing at The American Chestnut Foundation Meadowview Research Farm, Meadowview VA. Healthy cambial tissue was collected from the Chinese chestnut blight resistant genotypes 'Nanking' and 'Mahogany' growing at The American Chestnut Foundation Meadowview Research Farm, Meadowview VA. Canker tissues were collected from Chinese chestnut genotype 'Nanking' growing at Meadowview Research Farm. To create cankers, the stems of Chestnut trees were inoculated with the hypervirulent *Cryphonectria *strain EP155 as described by Hebard and collaborators [55]. Canker tissues were sampled 5 and 14 days post-inoculation and pooled before RNA preparation. All samples were collected in liquid nitrogen and frozen at -80°C until use.

### RNA preparation and cDNA library synthesis

Total RNA was prepared by the method of Chang and collaborators [56]. Three to five grams of frozen tissue were weighed, ground to a fine powder under liquid nitrogen, and dispersed in CTAB buffer. Following 2 chloroform extractions, RNA was precipitated with LiCl_2_, again extracted with chloroform and precipitated with ethanol. The resulting RNA pellet was resuspended in 40–100 μl of DEPC-treated water, and the quality was assessed with an Agilent Technologies 2100 Bioanalyzer (Agilent Technologies). Poly(A) RNA was then separated from total RNA using the Poly(A) Purist kit (Ambion) and the quality assessed with an Agilent Technologies 2100 Bioanalyzer (Agilent Technologies). cDNA was synthesized from the mRNA using the Just cDNA kit (Stratagene) using random hexamer primers provided with the kit to obtain better 5' to 3' coverage of transcripts than is possible using Poly(A) priming alone.

The resulting cDNA was used to construct a 454 library following the supplier's instructions (Roche Diagnostics). The sequencing was conducted at Penn State University using an FLX model 454 DNA sequencer (Roche Diagnostics).

### 454 library construction and sequencing

454 libraries were constructed as described previously [57]. In summary, cDNAs were sheared by nebulization to yield fragments approximately 500 bp in length. Adaptor sequences were ligated to fragmented cDNA, which were subsequently immobilized on beads. The DNA fragments were then denatured to yield a single stranded DNA library which was amplified by emulsion PCR for sequencing. Sequencing of the library was performed on a GS20 and an FLX model 454 DNA sequencer (454 Life Sciences). All raw 454 sequence data generated in this study is available at the Short Read Archive at the National Center for Biotechnology Information [24], specifically NCBI accession numbers SRX001799, SRX001800, SRX001801, SRX001804, RX001805, and SRX001806 (submission SRP000395).

### Transcript Assembly and analysis

The data from the 454 read sequences were assembled into transcript contigs using Newbler Assembler software (Roche). Reads from each library were assembled separately. The unigene (contigs and remaining unique singletons) sequences were annotated by query against the proteomes of *Arabidopsis *[58]) and *Populus *[59] and the predicted proteome for the blight fungus *Cryphonectria parasitica *[60] using Blastx (e-value cutoff of -10). The Gene Ontology (GO) (Consortium, 2008) system was used to summarize possible functional classifications of the unigenes via assignment of *Arabidopsis *gene identifiers with the strongest BLASTx alignments to the corresponding chestnut 454 reads. Comparison of the distribution of biological processes or molecular function obtained using Go annotation was done using GOstat program [26]. Comparison of gene expression between American chestnut canker and Chinese chestnut canker tissues as well as between canker and healthy stem tissues within each species was done using test developed by Dr. Claverie's team [27].

## Abbreviations

CAD: cinnamyl alcohol dehydrogenase; CCR: cinnamyl-CoA reductase; cDNA: complementary DNA; EST: expressed sequence tag; GO: Gene Ontology; Mb: megabases; NCBI: National Center for Biotechnology Information; nt: nucleotide; SAGE: Serial Analysis of Gene Expression.

## Authors' contributions

AB contributed to extracting RNA and making the 454 libraries, curated and analyzed the data, supervised the work SC and YZ, and wrote the paper. CS and YZ contributed to the bioinformatics analyses. DSD collected tissue samples, prepared RNA, cDNA and 454 libraries, and helped prepare the first draft of the manuscript. KB and WAP contributed to the RNA preparation and discussion of disease response gene candidates. NW manages all aspects of the Genomic Tool Development for the Fagaceae Project. RS is the Principle Investigator of the Genomic Tool Development for the Fagaceae Project and was responsible for oversight, budget, obtaining the funding for the project, and contributing advice at each step of the research. This work was conducted in the laboratory of JC, who initiated the research with American Chestnut Foundation funding, co-directs the 454 sequencing facility at Penn State, and contributed to the development of 454 sequencing protocols, evaluation and discussion of the results, and preparation of the manuscript.

## Supplementary Material

Additional File 1 **Genes more highly expressed in canker tissues than healthy stem tissues of American Chestnut.** Click here for file

Additional File 2 **Genes more highly expressed in canker tissues than in healthy stem tissues of Chinese Chestnut.** Click here for file

Additional File 3 **Disease and Defense Response Genes More Highly Expressed in Infected Tissues of Chinese Chestnut (CC) than in American Chestnut (AC).** Click here for file

Additional File 4 **Disease- and Defense- Response Genes More Highly Expressed in Infected Tissues of American Chestnut (AC) than in Chinese Chestnut (CC).** Click here for file

## References

[B1] Lang P, Dane F, Kubisiak TL, Huang H (2007). Molecular evidence for an Asian origin and a unique westward migration of species in the genus Castanea via Europe to North America. Mol Phylogenet Evol.

[B2] Griffin GJ (2000). Blight Control and Resoration of the American Chestnut. Journal of Forestry.

[B3] Keveer C (1953). Present compostion of some stands of the former oak-chestmut forest in southern Blue Ridge Mountains. Ecology.

[B4] Kubisiak TL, Hebarb CD, Nelson JZ, Bernatzky H, Huang S, Anagnostakis L, Doudrick RL (1997). Molecular Mapping of Resistance to Blight in an Interspecific Cross in the Genus *Castanea*. Phytopathology.

[B5] Freinkel S (2007). American Chestnut: The Life, Death, and Rebirth of a Perfect Tree.

[B6] Connors BJ, Maynard CA, Powell WA (2001). Expressed sequence tags from stem tissue of the American chestnut, *Castanea dentata*. Biotechnology Letters.

[B7] Roane MK, Griffin GJ, Elkins JR (1986). Chestnut Blight, Other Endothia Diseases and the Genus Endothia. Am Phytopathol Soc Monograph Series.

[B8] Brewer LG (1995). Ecology of Survival and Recovery from Blight in American Chestnut Trees (*Castanea dentata *(Marsh.) Borkh.) in Michigan. Bulletin of the Torrey Botanical Club.

[B9] Andrade GM, Merkle SA (2005). Enhancement of American chestnut somatic seedling production. Plant Cell Rep.

[B10] Milgroom MG, Cortesi P (2004). Biological control of chestnut blight with hypovirulence: a critical analysis. Annu Rev Phytopathol.

[B11] The American Chestnut Foundation. http://www.acf.org.

[B12] Fernando DD, Richards JL, Kikkert JR (2006). In vitro germination and transient GFP expression of American chestnut (Castanea dentata) pollen. Plant Cell Rep.

[B13] Polin LD, Liang H, Rothrock R, Nishii M, Diehl D, Newhouse A, Nairn CJ, Powell WA, Maynard CA (2006). Agrobacterium-mediated transformation of American chestnut (Castanea dentata (Marsh.) Borkh.) somatic embryos. Plant Cell Tissue and Organ Culture.

[B14] Merkle SA, Wiecko AT, Watson-Pauley BA (1991). Somatic embryogenesis in American Chestnut. Can J For Res.

[B15] Carraway DT, Merkle SA (1997). Plantlet regeneration fomr somatic embryos of American Chestnut. Can J For Res.

[B16] Xing Z, Powell WA, Maynard CA (1999). Development and germination of American chestnut somatic embryos. Plant Cell, Tissue and Organ Culture.

[B17] The Fagaceae Genomic Tools Project. http://www.fagaceae.org.

[B18] Margulies M, Egholm M, Altman WE, Attiya S, Bader JS, Bemben LA, Berka J, Braverman MS, Chen YJ, Chen Z (2005). Genome sequencing in microfabricated high-density picolitre reactors. Nature.

[B19] Weber AP, Weber KL, Carr K, Wilkerson C, Ohlrogge JB (2007). Sampling the Arabidopsis transcriptome with massively parallel pyrosequencing. Plant Physiol.

[B20] Redestig H, Weicht D, Selbig J, Hannah MA (2007). Transcription factor target prediction using multiple short expression time series from Arabidopsis thaliana. BMC Bioinformatics.

[B21] Ohtsu K, Smith MB, Emrich SJ, Borsuk LA, Zhou R, Chen T, Zhang X, Timmermans MC, Beck J, Buckner B (2007). Global gene expression analysis of the shoot apical meristem of maize (Zea mays L.). Plant J.

[B22] Emrich SJ, Barbazuk WB, Li L, Schnable PS (2007). Gene discovery and annotation using LCM-454 transcriptome sequencing. Genome Res.

[B23] Morozova O, Marra MA (2008). Applications of next-generation sequencing technologies in functional genomics. Genomics.

[B24] National Center for Biotechnology Information. http://www.ncbi.nlm.nih.gov.

[B25] Consortium GO (2008). The Gene Ontology project in 2008. Nucleic Acids Res.

[B26] Beissbarth T, Speed TP (2004). GOstat: find statistically overrepresented Gene Ontologies within a group of genes. Bioinformatics.

[B27] Audic S, Claverie JM (1997). The significance of digital gene expression profiles. Genome Res.

[B28] Carlson J, Leebens-Mack JH, Wall PK, Zahn LM, Mueller LA, Landherr LL, Hu Y, Ilut DC, Arrington JM, Choirean S, Becker A, Field D, Tanksley SD, Ma H, de Pamphilis CW (2006). EST database for early flower development in California poppy (Eschscholzia californica Cham., Papaveraceae) tags over 6,000 genes from a basal eudicot. Plant Mol Biol.

[B29] Lopez C, Jorge V, Piegu B, Mba C, Cortes D, Restrepo S, Soto M, Laudie M, Berger C, Cooke R (2004). A unigene catalogue of 5700 expressed genes in cassava. Plant Mol Biol.

[B30] Davies G, Henrissat B (1995). Structures and mechanisms of glycosyl hydrolases. Structure.

[B31] Talapatra S, Wagner JD, Thompson CB (2002). Elongation factor-1 alpha is a selective regulator of growth factor withdrawal and ER stress-induced apoptosis. Cell Death Differ.

[B32] Kim YC, Kim SY, Choi D, Ryu CM, Park JM (2008). Molecular characterization of a pepper C2 domain-containing SRC2 protein implicated in resistance against host and non-host pathogens and abiotic stresses. Planta.

[B33] Torres MA, Jones JD, Dangl JL (2005). Pathogen-induced, NADPH oxidase-derived reactive oxygen intermediates suppress spread of cell death in Arabidopsis thaliana. Nat Genet.

[B34] Kobae Y, Sekino T, Yoshioka H, Nakagawa T, Martinoia E, Maeshima M (2006). Loss of AtPDR8, a plasma membrane ABC transporter of Arabidopsis thaliana, causes hypersensitive cell death upon pathogen infection. Plant Cell Physiol.

[B35] Stukkens Y, Bultreys A, Grec S, Trombik T, Vanham D, Boutry M (2005). NpPDR1, a pleiotropic drug resistance-type ATP-binding cassette transporter from Nicotiana plumbaginifolia, plays a major role in plant pathogen defense. Plant Physiol.

[B36] Panikashvili D, Savaldi-Goldstein S, Mandel T, Yifhar T, Franke RB, Hofer R, Schreiber L, Chory J, Aharoni A (2007). The Arabidopsis DESPERADO/AtWBC11 transporter is required for cutin and wax secretion. Plant Physiol.

[B37] Sanchez-Fernandez R, Davies TG, Coleman JO, Rea PA (2001). The Arabidopsis thaliana ABC protein superfamily, a complete inventory. J Biol Chem.

[B38] Sibout R, Eudes A, Mouille G, Pollet B, Lapierre C, Jouanin L, Seguin A (2005). CINNAMYL ALCOHOL DEHYDROGENASE-C and -D are the primary genes involved in lignin biosynthesis in the floral stem of Arabidopsis. Plant Cell.

[B39] Qi X, Bakht S, Qin B, Leggett M, Hemmings A, Mellon F, Eagles J, Werck-Reichhart D, Schaller H, Lesot A (2006). A different function for a member of an ancient and highly conserved cytochrome P450 family: from essential sterols to plant defense. Proc Natl Acad Sci USA.

[B40] Kawasaki T, Hisako K, Nakatsubo T, Hasegawa K, Wakabayashi, Takahashi H, Umemura K, Umezawa T, Shimamoto (2006). Cinnamoyl-CoA reductase, a key enzyme in lignin biosynthesis, is an effector of small GTPase Rac in defense signaling in rice. PNAS.

[B41] Kawalleck P, Plesch G, Hahlbrock K, Somssich IE (1992). Induction by fungal elicitor of S-adenosyl-L-methionine synthetase and S-adenosyl-L-homocysteine hydrolase mRNAs in cultured cells and leaves of Petroselinum crispum. Proc Natl Acad Sci USA.

[B42] Wang G, Ding X, Yuan M, Qiu D, Li X, Xu C, Wang S (2006). Dual function of rice OsDR8 gene in disease resistance and thiamine accumulation. Plant Mol Biol.

[B43] Li X, Schuler MA, Berenbaum MR (2007). Molecular mechanisms of metabolic resistance to synthetic and natural xenobiotics. Annu Rev Entomol.

[B44] Chaturvedi R, Krothapalli K, Makandar R, Nandi A, Sparks AA, Roth MR, Welti R, Shah J (2008). Plastid omega3-fatty acid desaturase-dependent accumulation of a systemic acquired resistance inducing activity in petiole exudates of Arabidopsis thaliana is independent of jasmonic acid. Plant J.

[B45] Bovie C, Ongena M, Thonart P, Dommes J (2004). Cloning and expression analysis of cDNAs corresponding to genes activated in cucumber showing systemic acquired resistance after BTH treatment. BMC Plant Biol.

[B46] Brader G, Djamei A, Teige M, Palva ET, Hirt H (2007). The MAP kinase kinase MKK2 affects disease resistance in Arabidopsis. Mol Plant Microbe Interact.

[B47] Shoresh M, Gal-On A, Leibman D, Chet I (2006). Characterization of a mitogen-activated protein kinase gene from cucumber required for trichoderma-conferred plant resistance. Plant Physiol.

[B48] Yang Y, Klessig DF (1996). Isolation and characterization of a tobacco mosaic virus-inducible myb oncogene homolog from tobacco. Proc Natl Acad Sci USA.

[B49] Lee MW, Qi M, Yang Y (2001). A novel jasmonic acid-inducible rice myb gene associates with fungal infection and host cell death. Mol Plant Microbe Interact.

[B50] Vailleau F, Daniel X, Tronchet M, Montillet JL, Triantaphylides C, Roby D (2002). A R2R3-MYB gene, AtMYB30, acts as a positive regulator of the hypersensitive cell death program in plants in response to pathogen attack. Proc Natl Acad Sci USA.

[B51] Sugimoto K, Takeda S, Hirochika H (2000). MYB-related transcription factor NtMYB2 induced by wounding and elicitors is a regulator of the tobacco retrotransposon Tto1 and defense-related genes. Plant Cell.

[B52] Journot-Catalino N, Somssich IE, Roby D, Kroj T (2006). The transcription factors WRKY11 and WRKY17 act as negative regulators of basal resistance in Arabidopsis thaliana. Plant Cell.

[B53] Chen W, Provart NJ, Glazebrook J, Katagiri F, Chang HS, Eulgem T, Mauch F, Luan S, Zou G, Whitham SA (2002). Expression profile matrix of Arabidopsis transcription factor genes suggests their putative functions in response to environmental stresses. Plant Cell.

[B54] Marathe R, Guan Z, Anandalakshmi R, Zhao H, Dinesh-Kumar SP (2004). Study of Arabidopsis thaliana resistome in response to cucumber mosaic virus infection using whole genome microarray. Plant Mol Biol.

[B55] Hebard F, Griffin G, Elkins J (1984). Developmental histopathology of cankers incited by virulent and hypovirulent *Endothia parasitica *on susceptible and resistant chestnut trees. Phytopathology.

[B56] Chang S, Puryear J, Cairney J (1993). A simple and efficient method for isolating RNA from pine trees. Plant Mol Biol Rep.

[B57] Poinar HN, Schwarz C, Qi J, Shapiro B, Macphee RD, Buigues B, Tikhonov A, Huson DH, Tomsho LP, Auch A (2006). Metagenomics to paleogenomics: large-scale sequencing of mammoth DNA. Science.

[B58] The Arabidopsis Information Resource. http://www.arabidopsis.org.

[B59] Joint Genome Institute. http://www.jgi.doe.gov.

[B60] The predicted proteome for the blight fungus *Cryphonectria parasitica*. http://shake.jgi-psf.org/Crypa1/Crypa1.home.html.

