# Supplementary material for: Comparison of the transcriptomes of American chestnut (Castanea dentata) and Chinese chestnut (Castanea mollissima) in response to the chestnut blight infection
Source: BMC Plant Biol. 2009 May 9;9:51. doi: 10.1186/1471-2229-9-51 (PMC2688492; doi:10.1186/1471-2229-9-51)
Supplement: Additional File 1 — Genes more highly expressed in canker tissues than healthy stem tissues of American Chestnut. [file 1471-2229-9-51-S1.docx]

**Additional** **file 1. Genes more highly expressed in canker tissues than healthy stem tissues of American Chestnut. (*)** indicate significant differential expression at 95% confidence level.

| **Arabidopsis Accession #** | **# Canker Reads** | **% Canker Transcriptome** | **# Healthy Stem Reads** | **% Healthy Stem Transcriptome** | **Putative Function** |
| --- | --- | --- | --- | --- | --- |
| *AT1G76690.1 | 54 | 0.0417% | 1 | 0.0005% | 12-oxophytodienoate reductase (OPR2) |
| *AT4G34050.2 | 48 | 0.0371% | 1 | 0.0005% | caffeoyl-CoA 3-O-methyltransferase, putative |
| *AT1G62380.1 | 30 | 0.0232% | 1 | 0.0005% | 1-aminocyclopropane-1-carboxylate oxidase, putative / ACC oxidase, putative |
| *AT4G11650.1 | 25 | 0.0193% | 1 | 0.0005% | osmotin-like protein (OSM34) |
| *AT3G57490.1 | 22 | 0.0170% | 1 | 0.0005% | 40S ribosomal protein S2 (RPS2D) |
| *AT3G54420.1 | 18 | 0.0139% | 2 | 0.0011% | class IV chitinase (CHIV) |
| *AT5G36880.2 | 17 | 0.0131% | 2 | 0.0011% | similar to acyl-activating enzyme 17 (AAE17) |
| *AT1G76680.1 | 16 | 0.0124% | 1 | 0.0005% | 12-oxophytodienoate reductase (OPR1) |
| *AT4G10040.1 | 16 | 0.0124% | 2 | 0.0011% | cytochrome c, putative |
| *AT1G26880.1 | 15 | 0.0116% | 2 | 0.0011% | 60S ribosomal protein L34 (RPL34A) |
| *AT4G00100.1 | 14 | 0.0108% | 1 | 0.0005% | 40S ribosomal protein S13 (RPS13A) |
| *AT5G15520.1 | 13 | 0.0100% | 2 | 0.0011% | 40S ribosomal protein S19 (RPS19B) |
| *AT1G23820.1 | 13 | 0.0100% | 2 | 0.0011% | spermidine synthase 1 (SPDSYN1) / putrescine aminopropyltransferase 1 |
| *AT3G08900.1 | 13 | 0.0100% | 2 | 0.0011% | reversibly glycosylated polypeptide-3 (RGP3) |
| *AT5G13490.2 | 13 | 0.0100% | 2 | 0.0011% | similar to ADP, ATP carrier protein 1, mitochondrial / ADP/ATP translocase 1 |
| *AT1G60730.1 | 13 | 0.0100% | 1 | 0.0005% | aldo/keto reductase family protein |
| *AT4G37990.1 | 12 | 0.0093% | 1 | 0.0005% | mannitol dehydrogenase, putative (ELI3-2) |
| *AT3G09820.2 | 12 | 0.0093% | 1 | 0.0005% | adenosine kinase 1 (ADK1) / adenosine 5'-phosphotransferase 1 |
| *AT2G01250.1 | 11 | 0.0085% | 2 | 0.0011% | 60S ribosomal protein L7 (RPL7B) |
| *AT1G23290.1 | 11 | 0.0085% | 1 | 0.0005% | 60S ribosomal protein L27A (RPL27aB) |
| *AT5G62480.1 | 11 | 0.0085% | 1 | 0.0005% | glutathione S-transferase, putative \| chr5:25105944-25106792 REVERSE \| |
| *AT2G29420.1 | 11 | 0.0085% | 2 | 0.0011% | glutathione S-transferase, putative \| chr2:12625013-12625976 REVERSE \| |
| *AT3G16080.1 | 11 | 0.0085% | 2 | 0.0011% | 60S ribosomal protein L37 (RPL37C) |
| *AT1G73010.1 | 11 | 0.0085% | 1 | 0.0005% | expressed protein |
| *AT1G15950.1 | 11 | 0.0085% | 1 | 0.0005% | cinnamoyl-CoA reductase, putative |
| *AT2G16600.1 | 11 | 0.0085% | 1 | 0.0005% | peptidyl-prolyl cis-trans isomerase, cytosolic / cyclophilin / rotamase (ROC3) |
| *AT5G47770.1 | 10 | 0.0077% | 2 | 0.0011% | farnesyl pyrophosphate synthetase 1, mitochondrial (FPS1) / FPP synthetase 1 |
| *AT5G28060.1 | 10 | 0.0077% | 2 | 0.0011% | 40S ribosomal protein S24 (RPS24B), 40S ribosomal protein S19, Cyanophora paradoxa |
| *AT1G77940.1 | 10 | 0.0077% | 1 | 0.0005% | 60S ribosomal protein L30 (RPL30B) |
| *AT1G69530.2 | 10 | 0.0077% | 1 | 0.0005% | expansin, putative (EXP1) |
| *AT4G09320.1 | 9 | 0.0069% | 2 | 0.0011% | nucleoside diphosphate kinase 1 (NDK1) |
| *AT1G48830.2 | 9 | 0.0069% | 1 | 0.0005% | 40S ribosomal protein S7 (RPS7A) |
| *AT3G59970.3 | 9 | 0.0069% | 2 | 0.0011% | methylenetetrahydrofolate reductase 1 (MTHFR1) |
| *AT5G13080.1 | 9 | 0.0069% | 2 | 0.0011% | WRKY family transcription factor, WRKY DNA binding protein - Solanum tuberosum |
| *AT5G26667.3 | 9 | 0.0069% | 1 | 0.0005% | similar to uridylate kinase, putative |
| *AT1G16300.1 | 9 | 0.0069% | 2 | 0.0011% | glyceraldehyde 3-phosphate dehydrogenase, cytosolic, putative |
| *AT1G65220.1 | 9 | 0.0069% | 2 | 0.0011% | eIF4-gamma/eIF5/eIF2-epsilon domain-containing protein |
| *AT4G39330.1 | 8 | 0.0062% | 1 | 0.0005% | mannitol dehydrogenase, putative |
| *AT1G78870.3 | 8 | 0.0062% | 1 | 0.0005% | similar to ubiquitin-conjugating enzyme, putative |
| *AT5G60620.1 | 8 | 0.0062% | 2 | 0.0011% | phospholipid/glycerol acyltransferase family protein |
| *AT5G63180.1 | 8 | 0.0062% | 2 | 0.0011% | pectate lyase family protein |
| *AT3G60245.1 | 8 | 0.0062% | 1 | 0.0005% | 60S ribosomal protein L37a (RPL37aC) |
| *AT5G26340.1 | 8 | 0.0062% | 2 | 0.0011% | hexose transporter, putative |
| *AT4G01480.1 | 8 | 0.0062% | 2 | 0.0011% | inorganic pyrophosphatase, putative (soluble) / pyrophosphate phospho-hydrolase, putative / PPase, putative |
| *ATCG00120.1 | 8 | 0.0062% | 2 | 0.0011% | ATPase alpha subunit |
| vAT1G22360.2 | 8 | 0.0062% | 2 | 0.0011% | similar to UDP-glucoronosyl/UDP-glucosyl transferase family protein |
| *AT5G20000.1 | 8 | 0.0062% | 2 | 0.0011% | 26S proteasome AAA-ATPase subunit, putative |
| *AT2G22590.1 | 8 | 0.0062% | 1 | 0.0005% | glycosyltransferase family protein |
| *AT3G60100.1 | 7 | 0.0054% | 1 | 0.0005% | citrate synthase, mitochondrial, putative |
| *AT5G17380.1 | 7 | 0.0054% | 1 | 0.0005% | pyruvate decarboxylase family protein |
| *AT1G12010.1 | 7 | 0.0054% | 1 | 0.0005% | 1-aminocyclopropane-1-carboxylate oxidase, putative / ACC oxidase, putative |
| vAT1G26910.1 | 7 | 0.0054% | 2 | 0.0011% | 60S ribosomal protein L10 (RPL10B) |
| *AT4G11820.2 | 7 | 0.0054% | 2 | 0.0011% | hydroxymethylglutaryl-CoA synthase / HMG-CoA synthase / 3-hydroxy-3-methylglutaryl coenzyme A synthase |
| *AT5G05780.1 | 7 | 0.0054% | 2 | 0.0011% | 26S proteasome non-ATPase regulatory subunit 7, putative / 26S proteasome regulatory subunit S12, putative |
| *AT1G02620.1 | 7 | 0.0054% | 2 | 0.0011% | GTP-binding protein (SAR1A) |
| *AT3G49910.1 | 7 | 0.0054% | 1 | 0.0005% | 60S ribosomal protein L26 (RPL26A), 60S RIBOSOMAL PROTEIN L26, Brassica rapa |
| *AT5G15200.1 | 7 | 0.0054% | 1 | 0.0005% | 40S ribosomal protein S9 (RPS9B), 40S ribosomal protein S9, Chlamydomonas sp. |
| *AT4G23920.1 | 7 | 0.0054% | 1 | 0.0005% | UDP-glucose 4-epimerase, putative / UDP-galactose 4-epimerase, putative / Galactowaldenase, putative |
| *AT5G23740.1 | 7 | 0.0054% | 1 | 0.0005% | 40S ribosomal protein S11 (RPS11C) |
| *AT4G34200.1 | 7 | 0.0054% | 1 | 0.0005% | D-3-phosphoglycerate dehydrogenase, putative / 3-PGDH, putative |
| *AT3G52560.2 | 7 | 0.0054% | 1 | 0.0005% | ubiquitin-conjugating enzyme family protein |
| *AT1G78955.1 | 7 | 0.0054% | 1 | 0.0005% | beta-amyrin synthase, putative |
| *AT1G78370.1 | 7 | 0.0054% | 2 | 0.0011% | glutathione S-transferase, putative |
| *AT3G51000.1 | 6 | 0.0046% | 1 | 0.0005% | epoxide hydrolase, putative |
| *AT2G17500.4 | 6 | 0.0046% | 1 | 0.0005% | similar to auxin efflux carrier family protein |
| *AT5G13430.1 | 6 | 0.0046% | 1 | 0.0005% | ubiquinol-cytochrome C reductase iron-sulfur subunit, mitochondrial, putative / Rieske iron-sulfur protein |
| *AT1G12640.1 | 6 | 0.0046% | 1 | 0.0005% | membrane bound O-acyl transferase (MBOAT) family protein |
| *AT2G19590.1 | 6 | 0.0046% | 1 | 0.0005% | 1-aminocyclopropane-1-carboxylate oxidase, putative / ACC oxidase, putative, similar to ACC oxidase |
| *AT3G55920.1 | 6 | 0.0046% | 1 | 0.0005% | peptidyl-prolyl cis-trans isomerase, putative / cyclophilin, putative / rotamase, putative |
| *AT4G39220.1 | 6 | 0.0046% | 1 | 0.0005% | RER1A protein |
| AT5G18900.1 | 6 | 0.0046% | 2 | 0.0011% | oxidoreductase, 2OG-Fe(II) oxygenase family protein |
| *AT1G17880.1 | 6 | 0.0046% | 1 | 0.0005% | nascent polypeptide-associated complex (NAC) domain-containing protein / BTF3b-like transcription factor |
| *AT3G55380.1 | 6 | 0.0046% | 1 | 0.0005% | ubiquitin-conjugating enzyme 14 (UBC14), E2; UbcAT3 |
| *AT3G02780.1 | 6 | 0.0046% | 1 | 0.0005% | isopentenyl-diphosphate delta-isomerase II |
| *AT2G33120.2 | 6 | 0.0046% | 1 | 0.0005% | similar to synaptobrevin family protein |
| AT2G41840.1 | 6 | 0.0046% | 2 | 0.0011% | 40S ribosomal protein S2 (RPS2C) |
| *AT4G34870.1 | 6 | 0.0046% | 1 | 0.0005% | peptidyl-prolyl cis-trans isomerase / cyclophilin (CYP1) / rotamase |
| AT5G04800.4 | 6 | 0.0046% | 2 | 0.0011% | similar to 40S ribosomal protein S17 (RPS17A) |
| *AT5G02570.1 | 6 | 0.0046% | 1 | 0.0005% | histone H2B, putative |
| AT2G27020.1 | 6 | 0.0046% | 2 | 0.0011% | 20S proteasome alpha subunit G (PAG1) (PRC8) |
| *AT5G20940.1 | 6 | 0.0046% | 1 | 0.0005% | glycosyl hydrolase family 3 protein, beta-glucosidase, common nasturtium |
| AT1G77670.1 | 6 | 0.0046% | 2 | 0.0011% | aminotransferase class I and II family protein |
| AT1G43170.3 | 6 | 0.0046% | 2 | 0.0011% | similar to 60S ribosomal protein L3 (RPL3B) |
| AT3G02040.1 | 6 | 0.0046% | 2 | 0.0011% | glycerophosphoryl diester phosphodiesterase family protein |
| AT5G10960.1 | 6 | 0.0046% | 2 | 0.0011% | CCR4-NOT transcription complex protein, putative |
| *AT4G31985.1 | 6 | 0.0046% | 1 | 0.0005% | 60S ribosomal protein L39 (RPL39C) |
| *AT5G10330.2 | 6 | 0.0046% | 1 | 0.0005% | similar to histidinol-phosphate aminotransferase, putative |
| AT5G35680.2 | 6 | 0.0046% | 2 | 0.0011% | eukaryotic translation initiation factor 1A, putative / eIF-1A, putative / eIF-4C, putative |
| *AT4G26530.2 | 6 | 0.0046% | 1 | 0.0005% | similar to fructose-bisphosphate aldolase, cytoplasmic |
| AT5G11770.1 | 6 | 0.0046% | 2 | 0.0011% | NADH-ubiquinone oxidoreductase 20 kDa subunit, mitochondrial |
| AT1G48850.1 | 6 | 0.0046% | 2 | 0.0011% | chorismate synthase, putative / 5-enolpyruvylshikimate-3-phosphate phospholyase, putative |
| *AT4G14680.1 | 6 | 0.0046% | 1 | 0.0005% | sulfate adenylyltransferase 3 / ATP-sulfurylase 3 (APS3) |
| AT5G27850.1 | 5 | 0.0039% | 2 | 0.0011% | 60S ribosomal protein L18 (RPL18C), 60S ribosomal protein L18 |
| *AT5G13850.1 | 5 | 0.0039% | 1 | 0.0005% | nascent polypeptide-associated complex (NAC) domain-containing protein |
| *AT1G79210.1 | 5 | 0.0039% | 1 | 0.0005% | 20S proteasome alpha subunit B, putative |
| *AT5G28050.2 | 5 | 0.0039% | 1 | 0.0005% | similar to cytidine/deoxycytidylate deaminase family protein |
| *AT5G56710.1 | 5 | 0.0039% | 1 | 0.0005% | 60S ribosomal protein L31 (RPL31C) |
| AT5G56680.1 | 5 | 0.0039% | 2 | 0.0011% | asparaginyl-tRNA synthetase 1, cytoplasmic / asparagine-tRNA ligase 1 (SYNC1) |
| AT4G39730.1 | 5 | 0.0039% | 2 | 0.0011% | lipid-associated family protein |
| AT5G14590.1 | 5 | 0.0039% | 2 | 0.0011% | isocitrate dehydrogenase, putative / NADP+ isocitrate dehydrogenase, putative |
| *AT2G26660.1 | 5 | 0.0039% | 1 | 0.0005% | SPX (SYG1/Pho81/XPR1) domain-containing protein |
| *AT3G55500.1 | 5 | 0.0039% | 1 | 0.0005% | expansin, putative (EXP16) |
| AT2G45220.1 | 5 | 0.0039% | 2 | 0.0011% | pectinesterase family protein |
| *AT5G36130.1 | 5 | 0.0039% | 1 | 0.0005% | cytochrome P450 family |
| *AT3G49720.1 | 5 | 0.0039% | 1 | 0.0005% | expressed protein |
| *AT1G30270.2 | 5 | 0.0039% | 1 | 0.0005% | similar to CBL-interacting protein kinase 9 (CIPK9) |
| AT1G78340.1 | 5 | 0.0039% | 2 | 0.0011% | glutathione S-transferase, putative |
| *AT2G41530.1 | 5 | 0.0039% | 1 | 0.0005% | esterase, putative |
| *AT2G20840.1 | 5 | 0.0039% | 1 | 0.0005% | secretory carrier membrane protein (SCAMP) family protein |
| AT2G30050.1 | 5 | 0.0039% | 2 | 0.0011% | transducin family protein / WD-40 repeat family protein |
| AT3G24170.2 | 5 | 0.0039% | 2 | 0.0011% | similar to gluthatione reductase, chloroplast |
| *AT5G47760.1 | 5 | 0.0039% | 1 | 0.0005% | phosphoglycolate phosphatase, putative |
| *ATCG01050.1 | 5 | 0.0039% | 1 | 0.0005% | Translation data is not available for this gene |
| AT4G29340.1 | 5 | 0.0039% | 2 | 0.0011% | profilin 3 (PRO3) (PFN3) |
| *AT5G61240.1 | 5 | 0.0039% | 1 | 0.0005% | similar to leucine-rich repeat family protein |
| *AT5G60640.2 | 5 | 0.0039% | 1 | 0.0005% | thioredoxin family protein |
| *AT3G54250.1 | 5 | 0.0039% | 1 | 0.0005% | mevalonate diphosphate decarboxylase, putative |
| AT3G18080.1 | 5 | 0.0039% | 2 | 0.0011% | glycosyl hydrolase family 1 protein |
| *AT3G06300.1 | 5 | 0.0039% | 1 | 0.0005% | Encodes a prolyl-4 hydroxylase that can hydroxylate poly(L-proline)and other proline rich peptides |
| *AT3G13930.1 | 5 | 0.0039% | 1 | 0.0005% | dihydrolipoamide S-acetyltransferase, putative |
| AT5G20080.1 | 5 | 0.0039% | 2 | 0.0011% | NADH-cytochrome b5 reductase, putative |
| AT5G02450.1 | 5 | 0.0039% | 2 | 0.0011% | 60S ribosomal protein L36 (RPL36C), 60S ribosomal protein L36 |
| *AT3G18130.1 | 5 | 0.0039% | 1 | 0.0005% | guanine nucleotide-binding family protein / activated protein kinase C receptor (RACK1) |
| *AT4G27745.1 | 5 | 0.0039% | 1 | 0.0005% | similar to yippee family protein |
| *AT1G22170.1 | 5 | 0.0039% | 1 | 0.0005% | phosphoglycerate/bisphosphoglycerate mutase family protein |
| *AT1G78950.1 | 5 | 0.0039% | 1 | 0.0005% | beta-amyrin synthase, putative |
| *AT4G22570.1 | 5 | 0.0039% | 1 | 0.0005% | adenine phosphoribosyltransferase, putative |
| *AT1G21720.1 | 5 | 0.0039% | 1 | 0.0005% | 20S proteasome beta subunit C1 (PBC1) (PRCT) |
| AT5G10450.2 | 5 | 0.0039% | 2 | 0.0011% | similar to 14-3-3 protein GF14 kappa (GRF8) |
| ATCG01010.1 | 5 | 0.0039% | 2 | 0.0011% | Chloroplast encoded NADH dehydrogenase unit |
| AT3G57785.1 | 5 | 0.0039% | 2 | 0.0011% | expressed protein |
| *AT5G57020.1 | 4 | 0.0031% | 1 | 0.0005% | myristoyl-CoA:protein N-myristoyltransferase 1 (NMT1) |
| AT4G31180.2 | 4 | 0.0031% | 2 | 0.0011% | aspartyl-tRNA synthetase, putative / aspartate--tRNA ligase, putative |
| AT2G19770.1 | 4 | 0.0031% | 2 | 0.0011% | profilin 4 (PRO4) (PFN4) |
| AT1G02810.1 | 4 | 0.0031% | 2 | 0.0011% | pectinesterase family protein |
| AT4G08180.2 | 4 | 0.0031% | 2 | 0.0011% | oxysterol-binding family protein |
| AT1G14360.1 | 4 | 0.0031% | 2 | 0.0011% | UDP-galactose/UDP-glucose transporter, putative |
| AT4G02620.1 | 4 | 0.0031% | 2 | 0.0011% | vacuolar ATPase subunit F family protein |
| AT3G07890.1 | 4 | 0.0031% | 2 | 0.0011% | RabGAP/TBC domain-containing protein |
| AT1G02305.1 | 4 | 0.0031% | 2 | 0.0011% | cathepsin B-like cysteine protease, putative |
| AT3G28740.1 | 4 | 0.0031% | 1 | 0.0005% | cytochrome P450 family protein |
| AT4G24820.2 | 4 | 0.0031% | 1 | 0.0005% | 26S proteasome regulatory subunit, putative (RPN7) |
| AT2G44530.1 | 4 | 0.0031% | 2 | 0.0011% | ribose-phosphate pyrophosphokinase, putative / phosphoribosyl diphosphate synthetase, putative |
| AT3G02210.1 | 4 | 0.0031% | 1 | 0.0005% | phytochelatin synthetase family protein / COBRA cell expansion protein COBL3 |
| AT1G17170.1 | 4 | 0.0031% | 1 | 0.0005% | glutathione S-transferase, putative |
| AT2G32070.1 | 4 | 0.0031% | 2 | 0.0011% | CCR4-NOT transcription complex protein, putative |
| AT5G22780.1 | 4 | 0.0031% | 1 | 0.0005% | adaptin family protein |
| ATCG00430.1 | 4 | 0.0031% | 1 | 0.0005% | photosystem II G protein |
| AT1G31340.1 | 4 | 0.0031% | 2 | 0.0011% | ubiquitin family protein |
| AT2G17480.1 | 4 | 0.0031% | 1 | 0.0005% | seven transmembrane MLO family protein / MLO-like protein 8 (MLO8) |
| AT1G15370.1 | 4 | 0.0031% | 2 | 0.0011% | expressed protein |
| AT3G61790.1 | 4 | 0.0031% | 1 | 0.0005% | seven in absentia (SINA) family protein |
| AT3G58640.2 | 4 | 0.0031% | 2 | 0.0011% | protein kinase family protein |
| AT4G08150.1 | 4 | 0.0031% | 1 | 0.0005% | homeobox protein knotted-1 like 1 (KNAT1) |
| AT4G24220.1 | 4 | 0.0031% | 2 | 0.0011% | expressed protein, protein induced upon wounding |
| AT3G12860.1 | 4 | 0.0031% | 1 | 0.0005% | nucleolar protein Nop56, putative |
| AT4G21110.1 | 4 | 0.0031% | 1 | 0.0005% | G10 family protein |
| AT1G80930.1 | 4 | 0.0031% | 1 | 0.0005% | MIF4G domain-containing protein / MA3 domain-containing protein |
| ATCG01000.1 | 4 | 0.0031% | 2 | 0.0011% | hypothetical protein |
| AT5G58420.1 | 4 | 0.0031% | 2 | 0.0011% | 40S ribosomal protein S4 (RPS4D), ribosomal protein S4 |
| AT2G14720.2 | 4 | 0.0031% | 1 | 0.0005% | vacuolar sorting receptor, putative |
| AT5G47080.3 | 4 | 0.0031% | 2 | 0.0011% | similar to casein kinase II beta chain, putative |
| AT5G08030.1 | 4 | 0.0031% | 1 | 0.0005% | glycerophosphoryl diester phosphodiesterase family protein |
| AT5G56150.2 | 4 | 0.0031% | 1 | 0.0005% | ubiquitin-conjugating enzyme, putative |
| AT5G52840.1 | 4 | 0.0031% | 1 | 0.0005% | NADH-ubiquinone oxidoreductase-related |
| AT1G02130.1 | 4 | 0.0031% | 2 | 0.0011% | Ras-related protein (ARA-5) / small GTP-binding protein, putative |
| AT5G40810.1 | 4 | 0.0031% | 1 | 0.0005% | cytochrome c1, putative, cytochrome c1, heme protein, mitochondrial precursor |
| AT1G15120.1 | 4 | 0.0031% | 1 | 0.0005% | ubiquinol-cytochrome C reductase complex 7.8 kDa protein, putative / mitochondrial hinge protein, putative |
| ATCG01300.1 | 4 | 0.0031% | 1 | 0.0005% | One of two chloroplast genes that encode chloroplast ribosomal protein L23 |
| AT5G49910.1 | 4 | 0.0031% | 2 | 0.0011% | heat shock protein 70 / HSP70 (HSC70-7) |
| AT5G09920.1 | 4 | 0.0031% | 2 | 0.0011% | RNA polymerase II 15.9 kDa subunit (RPB15.9) |
| AT3G02870.2 | 4 | 0.0031% | 1 | 0.0005% | similar to inositol monophosphatase family protein |
| AT4G17170.1 | 4 | 0.0031% | 2 | 0.0011% | Rab2-like GTP-binding protein (RAB2) |
| AT4G09810.1 | 4 | 0.0031% | 2 | 0.0011% | transporter-related |
| AT3G12050.2 | 4 | 0.0031% | 2 | 0.0011% | Aha1 domain-containing protein |
| AT3G11670.1 | 4 | 0.0031% | 2 | 0.0011% | digalactosyldiacylglycerol synthase 1 (DGD1) |
| AT3G17020.1 | 4 | 0.0031% | 1 | 0.0005% | universal stress protein (USP) family protein |
| AT1G74210.1 | 4 | 0.0031% | 2 | 0.0011% | glycerophosphoryl diester phosphodiesterase family protein |
| AT4G21990.1 | 4 | 0.0031% | 2 | 0.0011% | 5'-adenylylsulfate reductase (APR3) / PAPS reductase homolog (PRH26) |
| AT2G34300.2 | 4 | 0.0031% | 2 | 0.0011% | similar to dehydration-responsive protein-related |
| ATCG01250.1 | 4 | 0.0031% | 2 | 0.0011% | NADH dehydrogenase ND2 |
| AT1G18540.1 | 4 | 0.0031% | 2 | 0.0011% | 60S ribosomal protein L6 (RPL6A) |
| AT3G49100.1 | 4 | 0.0031% | 1 | 0.0005% | signal recognition particle 9 kDa protein, putative / SRP9, putative |
| AT5G19550.1 | 4 | 0.0031% | 1 | 0.0005% | aspartate aminotransferase, cytoplasmic isozyme 1 / transaminase A (ASP2) |
| ATCG01090.1 | 4 | 0.0031% | 1 | 0.0005% | Encodes subunit of the chloroplast NAD(P)H dehydrogenase complex |
| AT2G39210.1 | 4 | 0.0031% | 2 | 0.0011% | nodulin family protein |
| AT1G72330.1 | 4 | 0.0031% | 1 | 0.0005% | alanine aminotransferase, putative |
| AT3G56070.1 | 4 | 0.0031% | 1 | 0.0005% | peptidyl-prolyl cis-trans isomerase, putative / cyclophilin, putative / rotamase, putative |
| AT2G29400.1 | 4 | 0.0031% | 1 | 0.0005% | serine/threonine protein phosphatase PP1 isozyme 1 (TOPP1) / phosphoprotein phosphatase 1 |
| AT5G47930.1 | 4 | 0.0031% | 1 | 0.0005% | 40S ribosomal protein S27 (RPS27D) |
| AT3G21230.1 | 4 | 0.0031% | 1 | 0.0005% | 4-coumarate--CoA ligase, putative / 4-coumaroyl-CoA synthase, putative (4CL) |
| ATCG00530.1 | 4 | 0.0031% | 2 | 0.0011% | hypothetical protein |
| AT1G03000.1 | 4 | 0.0031% | 2 | 0.0011% | AAA-type ATPase family protein |
| AT3G51370.2 | 4 | 0.0031% | 1 | 0.0005% | protein phosphatase 2C, putative / PP2C, putative |
| AT4G04610.1 | 4 | 0.0031% | 2 | 0.0011% | 5'-adenylylsulfate reductase (APR1) / PAPS reductase homolog (PRH19) |
| AT4G01870.1 | 4 | 0.0031% | 1 | 0.0005% | tolB protein-related |
| AT3G14290.1 | 4 | 0.0031% | 2 | 0.0011% | 20S proteasome alpha subunit E2 (PAE2) |
| AT1G63660.1 | 4 | 0.0031% | 1 | 0.0005% | GMP synthase (glutamine-hydrolyzing), putative / glutamine amidotransferase, putative |
| AT3G17820.1 | 4 | 0.0031% | 2 | 0.0011% | glutamine synthetase (GS1) |
| AT3G62290.1 | 4 | 0.0031% | 1 | 0.0005% | Gene encoding ADP-ribosylation factor and similar to other ARFs and ARF-like proteins |
| AT2G02470.1 | 4 | 0.0031% | 1 | 0.0005% | PHD finger family protein |
| AT5G16990.1 | 4 | 0.0031% | 2 | 0.0011% | NADP-dependent oxidoreductase, putative |
| AT1G65820.1 | 4 | 0.0031% | 1 | 0.0005% | microsomal glutathione s-transferase, putative |
| ATCG00380.1 | 4 | 0.0031% | 2 | 0.0011% | Chloroplast encoded ribosomal protein S4 |
| AT1G63000.1 | 4 | 0.0031% | 2 | 0.0011% | expressed protein |
| AT5G03540.1 | 4 | 0.0031% | 2 | 0.0011% | exocyst subunit EXO70 family protein |
| AT2G42790.1 | 3 | 0.0023% | 1 | 0.0005% | citrate synthase, glyoxysomal, putative |
| AT2G05840.2 | 3 | 0.0023% | 1 | 0.0005% | similar to 20S proteasome alpha subunit A1 (PAA1) (PRC1) |
| AT5G50370.1 | 3 | 0.0023% | 2 | 0.0011% | adenylate kinase, putative |
| AT4G11030.1 | 3 | 0.0023% | 1 | 0.0005% | long-chain-fatty-acid--CoA ligase, putative / long-chain acyl-CoA synthetase, putative |
| AT3G61430.1 | 3 | 0.0023% | 2 | 0.0011% | plasma membrane intrinsic protein 1A (PIP1A) / aquaporin PIP1.1 (PIP1.1) (AQ1) |
| AT3G05820.1 | 3 | 0.0023% | 1 | 0.0005% | beta-fructofuranosidase, putative / invertase, putative / saccharase, putative / beta-fructosidase, putative |
| AT1G50370.1 | 3 | 0.0023% | 2 | 0.0011% | serine/threonine protein phosphatase, putative |
| AT5G47720.5 | 3 | 0.0023% | 1 | 0.0005% | similar to acetyl-CoA C-acyltransferase, putative / 3-ketoacyl-CoA thiolase, putative |
| AT5G10240.2 | 3 | 0.0023% | 2 | 0.0011% | similar to asparagine synthetase 1 (glutamine-hydrolyzing) |
| AT1G04430.1 | 3 | 0.0023% | 1 | 0.0005% | dehydration-responsive protein-related |
| AT1G59710.1 | 3 | 0.0023% | 1 | 0.0005% | expressed protein |
| AT1G44750.3 | 3 | 0.0023% | 1 | 0.0005% | similar to purine permease family protein |
| AT5G39840.1 | 3 | 0.0023% | 2 | 0.0011% | ATP-dependent RNA helicase, mitochondrial, putative |
| AT1G11330.1 | 3 | 0.0023% | 2 | 0.0011% | S-locus lectin protein kinase family protein |
| AT2G09990.1 | 3 | 0.0023% | 2 | 0.0011% | 40S ribosomal protein S16 (RPS16A) |
| AT5G41520.1 | 3 | 0.0023% | 1 | 0.0005% | 40S ribosomal protein S10 (RPS10B) |
| AT3G22320.1 | 3 | 0.0023% | 2 | 0.0011% | DNA-directed RNA polymerase, putative |
| AT2G46280.2 | 3 | 0.0023% | 1 | 0.0005% | eukaryotic translation initiation factor 3 subunit 2 |
| AT1G74380.1 | 3 | 0.0023% | 2 | 0.0011% | galactosyl transferase GMA12/MNN10 family protein |
| AT5G52470.1 | 3 | 0.0023% | 2 | 0.0011% | fibrillarin 1 (FBR1) (FIB1) (SKIP7) |
| AT4G36690.2 | 3 | 0.0023% | 1 | 0.0005% | U2 snRNP auxiliary factor large subunit, putative |
| AT3G54700.1 | 3 | 0.0023% | 1 | 0.0005% | phosphate transporter, putative |
| AT2G20470.1 | 3 | 0.0023% | 1 | 0.0005% | protein kinase, putative |
| AT5G18400.2 | 3 | 0.0023% | 1 | 0.0005% | expressed protein |
| AT4G05390.1 | 3 | 0.0023% | 1 | 0.0005% | ferredoxin--NADP(+) reductase, putative / adrenodoxin reductase, putative |
| AT4G21940.1 | 3 | 0.0023% | 2 | 0.0011% | calcium-dependent protein kinase, putative / CDPK, putative |
| AT1G73780.1 | 3 | 0.0023% | 1 | 0.0005% | protease inhibitor/seed storage/lipid transfer protein (LTP) family protein |
| AT5G50310.1 | 3 | 0.0023% | 1 | 0.0005% | kelch repeat-containing protein |
| AT2G32720.1 | 3 | 0.0023% | 1 | 0.0005% | cytochrome b5, putative |
| AT3G10950.1 | 3 | 0.0023% | 2 | 0.0011% | 60S ribosomal protein L37a (RPL37aB) |
| AT3G16780.1 | 3 | 0.0023% | 1 | 0.0005% | 60S ribosomal protein L19 (RPL19B) |
| AT4G23900.1 | 3 | 0.0023% | 1 | 0.0005% | nucleoside diphosphate kinase 4 (NDK4) |
| AT2G26830.1 | 3 | 0.0023% | 1 | 0.0005% | choline/ethanolamine kinase family protein |
| AT2G41680.1 | 3 | 0.0023% | 1 | 0.0005% | thioredoxin reductase, putative / NADPH-dependent thioredoxin reductase, putative |
| AT3G10920.1 | 3 | 0.0023% | 2 | 0.0011% | superoxide dismutase (Mn), mitochondrial (SODA) / manganese superoxide dismutase (MSD1) |
| AT4G26910.1 | 3 | 0.0023% | 2 | 0.0011% | 2-oxoacid dehydrogenase family protein |
| AT4G19230.2 | 3 | 0.0023% | 2 | 0.0011% | cytochrome P450 family protein, cytochrome P450 |
| AT2G31750.1 | 3 | 0.0023% | 1 | 0.0005% | UDP-glucoronosyl/UDP-glucosyl transferase family protein |
| AT5G14200.3 | 3 | 0.0023% | 1 | 0.0005% | similar to 3-isopropylmalate dehydrogenase, chloroplast, putative |
| AT4G31550.2 | 3 | 0.0023% | 2 | 0.0011% | WRKY family transcription factor |
| AT2G01930.2 | 3 | 0.0023% | 1 | 0.0005% | expressed protein |
| AT5G35790.1 | 3 | 0.0023% | 1 | 0.0005% | Encodes a plastidic glucose-6-phosphate dehydrogenase |
| AT3G52730.1 | 3 | 0.0023% | 2 | 0.0011% | ubiquinol-cytochrome C reductase UQCRX/QCR9-like family protein |
| AT3G13540.1 | 3 | 0.0023% | 2 | 0.0011% | myb family transcription factor |
| AT5G22300.1 | 3 | 0.0023% | 1 | 0.0005% | nitrilase 4 (NIT4) |
| AT1G34370.3 | 3 | 0.0023% | 1 | 0.0005% | similar to zinc finger (C2H2 type) family protein |
| AT1G05500.1 | 3 | 0.0023% | 2 | 0.0011% | C2 domain-containing protein |
| AT5G62530.1 | 3 | 0.0023% | 1 | 0.0005% | delta-1-pyrroline-5-carboxylate dehydrogenase (P5CDH) |
| AT1G77130.1 | 3 | 0.0023% | 1 | 0.0005% | glycogenin glucosyltransferase (glycogenin)-related |
| AT2G36580.1 | 3 | 0.0023% | 2 | 0.0011% | pyruvate kinase, putative |
| AT4G01810.1 | 3 | 0.0023% | 2 | 0.0011% | protein transport protein-related |
| AT4G13170.1 | 3 | 0.0023% | 1 | 0.0005% | 60S ribosomal protein L13A (RPL13aC), ribosomal protein L13a -Lupinus luteus |
| AT5G20410.1 | 3 | 0.0023% | 1 | 0.0005% | 1,2-diacylglycerol 3-beta-galactosyltransferase, putative / monogalactosyldiacylglycerol synthase, putative |
| AT5G53530.1 | 3 | 0.0023% | 2 | 0.0011% | vacuolar protein sorting-associated protein 26, putative / VPS26, putative |
| AT2G48020.1 | 3 | 0.0023% | 1 | 0.0005% | sugar transporter, putative |
| AT5G64370.1 | 3 | 0.0023% | 1 | 0.0005% | PYD3 encodes a beta-ureidopropionase |
| AT5G48880.3 | 3 | 0.0023% | 1 | 0.0005% | similar to acetyl-CoA C-acyltransferase, putative / 3-ketoacyl-CoA thiolase, putative |
| AT2G32830.1 | 3 | 0.0023% | 2 | 0.0011% | inorganic phosphate transporter (PHT5) |
| AT3G62560.1 | 3 | 0.0023% | 2 | 0.0011% | GTP-binding protein, putative |
| AT4G28510.1 | 3 | 0.0023% | 2 | 0.0011% | prohibitin, putative |
| AT4G26300.1 | 3 | 0.0023% | 1 | 0.0005% | arginyl-tRNA synthetase, putative / arginine--tRNA ligase, putative |
| AT2G02010.1 | 3 | 0.0023% | 1 | 0.0005% | glutamate decarboxylase, putative |
| AT1G71697.1 | 3 | 0.0023% | 1 | 0.0005% | choline kinase, putative |
| AT5G26830.1 | 3 | 0.0023% | 1 | 0.0005% | threonyl-tRNA synthetase / threonine--tRNA ligase (THRRS) |
| AT3G17240.3 | 3 | 0.0023% | 2 | 0.0011% | dihydrolipoamide dehydrogenase 2, mitochondrial / lipoamide dehydrogenase 2 (MTLPD2) |
| AT1G48630.1 | 3 | 0.0023% | 1 | 0.0005% | guanine nucleotide-binding family protein / activated protein kinase C receptor, putative / RACK, putative |
| AT1G10430.1 | 3 | 0.0023% | 1 | 0.0005% | serine/threonine protein phosphatase PP2A-1 catalytic subunit (PP2A1) |
| AT1G22840.1 | 3 | 0.0023% | 1 | 0.0005% | cytochrome c, putative |
| AT2G11810.1 | 3 | 0.0023% | 1 | 0.0005% | 1,2-diacylglycerol 3-beta-galactosyltransferase, putative / monogalactosyldiacylglycerol synthase |
| AT5G14550.2 | 3 | 0.0023% | 1 | 0.0005% | expressed protein |
| AT5G37850.1 | 3 | 0.0023% | 1 | 0.0005% | pfkB-type carbohydrate kinase family protein |
| AT1G78870.2 | 3 | 0.0023% | 1 | 0.0005% | ubiquitin-conjugating enzyme, putative |
| AT4G34700.1 | 3 | 0.0023% | 2 | 0.0011% | complex 1 family protein / LVR family protein |
| AT2G30970.1 | 3 | 0.0023% | 1 | 0.0005% | aspartate aminotransferase, mitochondrial / transaminase A (ASP1) |
| AT5G43010.1 | 3 | 0.0023% | 2 | 0.0011% | 26S proteasome AAA-ATPase subunit (RPT4a) |
| AT2G17360.1 | 3 | 0.0023% | 1 | 0.0005% | 40S ribosomal protein S4 (RPS4A) |
| AT5G63870.1 | 3 | 0.0023% | 2 | 0.0011% | serine/threonine protein phosphatase (PP7) |
| AT5G13280.1 | 3 | 0.0023% | 2 | 0.0011% | aspartate kinase |
| AT3G20800.1 | 3 | 0.0023% | 2 | 0.0011% | rcd1-like cell differentiation protein, putative |
| AT3G55620.1 | 3 | 0.0023% | 1 | 0.0005% | eukaryotic translation initiation factor 6, putative / eIF-6, putative |
| AT1G07470.1 | 3 | 0.0023% | 2 | 0.0011% | transcription factor IIA large subunit, putative / TFIIA large subunit, putative |
| AT4G30680.1 | 3 | 0.0023% | 1 | 0.0005% | MA3 domain-containing protein |
| AT1G23210.1 | 3 | 0.0023% | 1 | 0.0005% | glycosyl hydrolase family 9 protein |
| AT4G26110.1 | 3 | 0.0023% | 2 | 0.0011% | nucleosome assembly protein (NAP), putative |
| AT1G29690.1 | 3 | 0.0023% | 1 | 0.0005% | protein containing a domain with significant homology to the membrane attack complex and perforin domain |
| AT2G22480.1 | 3 | 0.0023% | 2 | 0.0011% | phosphofructokinase family protein |
| AT4G27720.1 | 3 | 0.0023% | 2 | 0.0011% | expressed protein |
| AT3G20290.2 | 3 | 0.0023% | 2 | 0.0011% | similar to calcium-binding EF hand family protein |
| AT1G53990.1 | 3 | 0.0023% | 1 | 0.0005% | GDSL-motif lipase/hydrolase family protein |
| AT5G49555.1 | 3 | 0.0023% | 1 | 0.0005% | amine oxidase-related |
| AT1G05560.1 | 3 | 0.0023% | 1 | 0.0005% | UDP-glucose transferase (UGT75B2) |
| AT1G30620.2 | 3 | 0.0023% | 1 | 0.0005% | similar to NAD-dependent epimerase/dehydratase family protein |
| AT4G30950.1 | 3 | 0.0023% | 2 | 0.0011% | omega-6 fatty acid desaturase, chloroplast (FAD6) (FADC) |
| AT5G54760.1 | 3 | 0.0023% | 2 | 0.0011% | eukaryotic translation initiation factor SUI1, putative |
| AT3G23340.1 | 3 | 0.0023% | 2 | 0.0011% | casein kinase, putative |
| AT4G27020.1 | 3 | 0.0023% | 2 | 0.0011% | expressed protein |
| AT3G62720.2 | 3 | 0.0023% | 1 | 0.0005% | similar to galactosyl transferase GMA12/MNN10 family protein |
| AT1G04690.1 | 3 | 0.0023% | 2 | 0.0011% | potassium channel protein, putative |
| AT4G00400.1 | 3 | 0.0023% | 2 | 0.0011% | phospholipid/glycerol acyltransferase family protein |
| AT4G01690.1 | 3 | 0.0023% | 2 | 0.0011% | protoporphyrinogen oxidase (PPOX) |
| AT3G04600.2 | 3 | 0.0023% | 1 | 0.0005% | tRNA synthetase class I (W and Y) family protein |
| AT4G10080.1 | 3 | 0.0023% | 1 | 0.0005% | expressed protein |
| AT1G51540.1 | 3 | 0.0023% | 1 | 0.0005% | kelch repeat-containing protein |
| AT3G09560.1 | 3 | 0.0023% | 2 | 0.0011% | lipin family protein |
| AT2G04280.1 | 3 | 0.0023% | 2 | 0.0011% | expressed protein |
| AT5G37180.1 | 3 | 0.0023% | 2 | 0.0011% | sucrose synthase, putative / sucrose-UDP glucosyltransferase, putative |
| AT1G22540.1 | 3 | 0.0023% | 2 | 0.0011% | proton-dependent oligopeptide transport (POT) family protein |
| AT5G52450.1 | 3 | 0.0023% | 1 | 0.0005% | MATE efflux protein-related |
| AT5G64640.1 | 3 | 0.0023% | 2 | 0.0011% | pectinesterase family protein |
| AT1G19440.1 | 3 | 0.0023% | 1 | 0.0005% | very-long-chain fatty acid condensing enzyme, putative |
| AT1G54220.2 | 3 | 0.0023% | 1 | 0.0005% | similar to dihydrolipoamide S-acetyltransferase, putative |
| AT1G77440.2 | 3 | 0.0023% | 1 | 0.0005% | similar to 20S proteasome beta subunit C1 (PBC1) (PRCT) |
| AT5G06580.1 | 3 | 0.0023% | 1 | 0.0005% | FAD linked oxidase family protein |
| AT2G37640.1 | 3 | 0.0023% | 2 | 0.0011% | expansin, putative (EXP3) |
| AT2G27190.1 | 3 | 0.0023% | 2 | 0.0011% | iron(III)-zinc(II) purple acid phosphatase (PAP12) |
| AT1G27450.2 | 3 | 0.0023% | 2 | 0.0011% | adenine phosphoribosyltransferase 1 (APT1) |
| AT1G06020.1 | 3 | 0.0023% | 1 | 0.0005% | pfkB-type carbohydrate kinase family protein |
| AT5G41950.1 | 3 | 0.0023% | 2 | 0.0011% | expressed protein |
| AT1G74910.2 | 3 | 0.0023% | 1 | 0.0005% | ADP-glucose pyrophosphorylase family protein |
| AT1G65960.1 | 3 | 0.0023% | 1 | 0.0005% | similar to glutamate decarboxylase 1 (GAD 1) |
| AT1G56720.2 | 3 | 0.0023% | 2 | 0.0011% | protein kinase family protein |
| AT1G02560.1 | 3 | 0.0023% | 1 | 0.0005% | ATP-dependent Clp protease proteolytic subunit (ClpP1) |
| AT4G31780.2 | 3 | 0.0023% | 1 | 0.0005% | 1,2-diacylglycerol 3-beta-galactosyltransferase, putative / monogalactosyldiacylglycerol synthase, |
| AT1G56700.1 | 3 | 0.0023% | 2 | 0.0011% | pyrrolidone-carboxylate peptidase family protein |
| AT1G14510.1 | 3 | 0.0023% | 2 | 0.0011% | PHD finger family protein |
