# Supplementary material for: Comparison of the transcriptomes of American chestnut (Castanea dentata) and Chinese chestnut (Castanea mollissima) in response to the chestnut blight infection
Source: BMC Plant Biol. 2009 May 9;9:51. doi: 10.1186/1471-2229-9-51 (PMC2688492; doi:10.1186/1471-2229-9-51)
Supplement: Additional File 2 — Genes more highly expressed in canker tissues than in healthy stem tissues of Chinese Chestnut. [file 1471-2229-9-51-S2.docx]

**Additional file 2. Genes more highly expressed in canker tissues than in healthy stem tissues of Chinese Chestnut. (*)** indicate significant differential expression at 95% confidence level.

| **Arabidopsis Accession #** | **# Canker Reads** | **% Canker Transcriptome** | **# Healthy Stems Reads** | **% Healthy Stem Transcriptome** | **Function (Annotation in Arabidopsis)** |
| --- | --- | --- | --- | --- | --- |
| *AT1G62380.1 | 35 | 0.0149% | 1 | 0.0002% | 1-aminocyclopropane-1-carboxylate oxidase, putative / ACC oxidase, putative |
| *AT1G26630.1 | 15 | 0.0064% | 1 | 0.0002% | eukaryotic translation initiation factor 5A, putative / eIF-5A, putative |
| *AT3G13445.2 | 13 | 0.0055% | 2 | 0.0004% | similar to transcription initiation factor IID-2 (TFIID-2) / TATA-box factor 2 / TATA sequence-binding protein 2 |
| *AT1G76690.1 | 11 | 0.0047% | 1 | 0.0002% | 12-oxophytodienoate reductase (OPR2) |
| *AT3G07110.1 | 11 | 0.0047% | 1 | 0.0002% | 60S ribosomal protein L13A (RPL13aA) |
| *AT5G59160.3 | 11 | 0.0047% | 2 | 0.0004% | similar to serine/threonine protein phosphatase PP1 isozyme 5 (TOPP5) / phosphoprotein phosphatase 1 |
| *AT2G16430.2 | 10 | 0.0042% | 2 | 0.0004% | purple acid phosphatase (PAP10) |
| *AT1G62510.1 | 9 | 0.0038% | 2 | 0.0004% | protease inhibitor/seed storage/lipid transfer protein (LTP) family protein |
| *AT5G36130.1 | 9 | 0.0038% | 1 | 0.0002% | cytochrome P450 family |
| *AT5G35590.1 | 9 | 0.0038% | 1 | 0.0002% | 20S proteasome alpha subunit A1 (PAA1) (PRC1) |
| *AT2G38710.2 | 8 | 0.0034% | 2 | 0.0004% | similar to DUF51 family protein |
| *AT3G58750.1 | 8 | 0.0034% | 1 | 0.0002% | citrate synthase, glyoxysomal, putative |
| *AT5G13080.1 | 7 | 0.0030% | 1 | 0.0002% | WRKY family transcription factor, WRKY DNA binding protein - Solanum tuberosum |
| *AT5G37980.1 | 7 | 0.0030% | 1 | 0.0002% | NADP-dependent oxidoreductase, putative |
| *AT1G17880.1 | 7 | 0.0030% | 1 | 0.0002% | nascent polypeptide-associated complex (NAC) domain-containing protein / BTF3b-like transcription factor |
| *AT4G19230.2 | 7 | 0.0030% | 1 | 0.0002% | cytochrome P450 family protein, cytochrome P450 |
| *AT4G19120.2 | 7 | 0.0030% | 2 | 0.0004% | early-responsive to dehydration stress protein (ERD3) |
| *AT1G78370.1 | 7 | 0.0030% | 2 | 0.0004% | glutathione S-transferase, putative |
| *AT5G27770.1 | 7 | 0.0030% | 1 | 0.0002% | 60S ribosomal protein L22 (RPL22C), ribosomal protein L22 (cytosolic), Rattus norvegicus |
| *AT3G07890.1 | 7 | 0.0030% | 1 | 0.0002% | RabGAP/TBC domain-containing protein |
| *AT5G62480.1 | 6 | 0.0025% | 2 | 0.0004% | glutathione S-transferase, putative |
| *AT3G57520.2 | 6 | 0.0025% | 1 | 0.0002% | alkaline alpha galactosidase, putative |
| *AT4G17370.1 | 6 | 0.0025% | 1 | 0.0002% | oxidoreductase family protein |
| *AT5G01460.1 | 6 | 0.0025% | 1 | 0.0002% | LMBR1 integral membrane family protein |
| *AT3G15980.3 | 6 | 0.0025% | 2 | 0.0004% | coatomer protein complex, subunit beta 2 (beta prime), putative |
| *AT3G47860.1 | 6 | 0.0025% | 1 | 0.0002% | apolipoprotein D-related |
| *AT5G24420.1 | 6 | 0.0025% | 1 | 0.0002% | glucosamine/galactosamine-6-phosphate isomerase-related |
| *AT1G72330.1 | 6 | 0.0025% | 2 | 0.0004% | alanine aminotransferase, putative |
| *AT5G47930.1 | 6 | 0.0025% | 2 | 0.0004% | 40S ribosomal protein S27 (RPS27D) |
| *AT5G43780.1 | 6 | 0.0025% | 2 | 0.0004% | sulfate adenylyltransferase 4 / ATP-sulfurylase 4 (APS4) |
| *AT3G26340.1 | 6 | 0.0025% | 2 | 0.0004% | 20S proteasome beta subunit E, putative |
| *AT1G01820.1 | 6 | 0.0025% | 2 | 0.0004% | peroxisomal biogenesis factor 11 family protein / PEX11 family protein |
| *AT3G14790.1 | 6 | 0.0025% | 2 | 0.0004% | NAD-dependent epimerase/dehydratase family protein |
| *AT5G22020.1 | 6 | 0.0025% | 2 | 0.0004% | strictosidine synthase family protein |
| *AT5G41360.1 | 6 | 0.0025% | 1 | 0.0002% | Arabidopsis thaliana has duplicated XPB gene (AtXPB1 and AtXPB2, with high similarity to each other) |
| *AT5G46290.2 | 6 | 0.0025% | 1 | 0.0002% | similar to 3-ketoacyl-ACP synthase, putative |
| *AT4G08180.2 | 5 | 0.0021% | 2 | 0.0004% | oxysterol-binding family protein |
| *AT5G47080.3 | 5 | 0.0021% | 2 | 0.0004% | similar to casein kinase II beta chain, putative |
| *AT1G77130.1 | 5 | 0.0021% | 2 | 0.0004% | glycogenin glucosyltransferase (glycogenin)-related |
| *AT5G17760.1 | 5 | 0.0021% | 1 | 0.0002% | AAA-type ATPase family protein |
| *AT2G30970.1 | 5 | 0.0021% | 1 | 0.0002% | aspartate aminotransferase, mitochondrial / transaminase A (ASP1) |
| *AT1G52280.1 | 5 | 0.0021% | 2 | 0.0004% | Ras-related GTP-binding protein, putative |
| *AT3G13930.1 | 5 | 0.0021% | 2 | 0.0004% | dihydrolipoamide S-acetyltransferase, putative |
| *AT1G12010.1 | 5 | 0.0021% | 2 | 0.0004% | 1-aminocyclopropane-1-carboxylate oxidase, putative / ACC oxidase, putativ |
| *AT5G03520.2 | 5 | 0.0021% | 1 | 0.0002% | similar to Ras-related GTP-binding protein, putative |
| *AT1G09400.1 | 5 | 0.0021% | 1 | 0.0002% | 12-oxophytodienoate reductase, putative |
| *AT4G14305.1 | 5 | 0.0021% | 2 | 0.0004% | similar to peroxisomal membrane protein 22 kDa (PMP22) |
| *AT1G75060.1 | 5 | 0.0021% | 2 | 0.0004% | expressed protein |
| *AT3G01420.1 | 5 | 0.0021% | 1 | 0.0002% | pathogen-responsive alpha-dioxygenase, putative |
| *AT2G45220.1 | 5 | 0.0021% | 1 | 0.0002% | pectinesterase family protein |
| *AT3G17820.1 | 5 | 0.0021% | 1 | 0.0002% | glutamine synthetase (GS1) |
| AT2G27820.1 | 5 | 0.0021% | 2 | 0.0004% | prephenate dehydratase family protein |
| *AT5G23120.1 | 5 | 0.0021% | 1 | 0.0002% | photosystem II stability/assembly factor, chloroplast (HCF136) |
| *AT5G42990.1 | 5 | 0.0021% | 1 | 0.0002% | ubiquitin-conjugating enzyme 18 (UBC18), E2 |
| *AT4G09510.1 | 5 | 0.0021% | 1 | 0.0002% | beta-fructofuranosidase, putative / invertase, putative / saccharase, putative / beta-fructosidase, putative |
| *AT4G22720.2 | 4 | 0.0017% | 1 | 0.0002% | glycoprotease M22 family protein |
| AT1G80670.1 | 4 | 0.0017% | 2 | 0.0004% | transducin family protein / WD-40 repeat family protein |
| AT3G06790.2 | 4 | 0.0017% | 2 | 0.0004% | plastid developmental protein DAG, putative |
| AT4G22460.1 | 4 | 0.0017% | 2 | 0.0004% | protease inhibitor/seed storage/lipid transfer protein (LTP) family protein |
| AT5G24580.2 | 4 | 0.0017% | 2 | 0.0004% | copper-binding family protein |
| *AT5G54810.1 | 4 | 0.0017% | 1 | 0.0002% | tryptophan synthase, beta subunit 1 (TSB1) |
| AT3G19590.1 | 4 | 0.0017% | 2 | 0.0004% | WD-40 repeat family protein / mitotic checkpoint protein, putative |
| AT4G11240.1 | 4 | 0.0017% | 2 | 0.0004% | serine/threonine protein phosphatase PP1 isozyme 6 (PP1BG) (TOPP6) |
| AT1G80780.2 | 4 | 0.0017% | 2 | 0.0004% | CCR4-NOT transcription complex protein, putative |
| *AT1G07830.1 | 4 | 0.0017% | 1 | 0.0002% | ribosomal protein L29 family protein |
| AT3G11040.1 | 4 | 0.0017% | 2 | 0.0004% | glycosyl hydrolase family 85 protein |
| AT5G14200.3 | 4 | 0.0017% | 2 | 0.0004% | similar to 3-isopropylmalate dehydrogenase, chloroplast, putative |
| *AT5G66560.1 | 4 | 0.0017% | 1 | 0.0002% | phototropic-responsive NPH3 family protein |
| AT4G07390.1 | 4 | 0.0017% | 2 | 0.0004% | PQ-loop repeat family protein / transmembrane family protein |
| AT4G21100.1 | 4 | 0.0017% | 2 | 0.0004% | UV-damaged DNA-binding protein, putative |
| *AT5G66020.1 | 4 | 0.0017% | 1 | 0.0002% | Encodes a phosphoinositide phosphatase that modulates cellular phosphoinositide levels. |
| AT5G06720.1 | 4 | 0.0017% | 2 | 0.0004% | peroxidase, putative |
| AT1G52870.2 | 4 | 0.0017% | 2 | 0.0004% | peroxisomal membrane protein-related |
| AT4G15940.1 | 4 | 0.0017% | 2 | 0.0004% | fumarylacetoacetate hydrolase family protein |
| *AT2G42910.1 | 4 | 0.0017% | 1 | 0.0002% | ribose-phosphate pyrophosphokinase 4 / phosphoribosyl diphosphate synthetase 4 (PRS4) |
| AT3G54660.1 | 4 | 0.0017% | 2 | 0.0004% | gluthatione reductase, chloroplast |
| AT2G36310.1 | 4 | 0.0017% | 2 | 0.0004% | inosine-uridine preferring nucleoside hydrolase family protein |
| AT3G02580.1 | 4 | 0.0017% | 2 | 0.0004% | delta 7-sterol-C5-desaturase (STE1) |
| AT1G05805.1 | 4 | 0.0017% | 2 | 0.0004% | basic helix-loop-helix (bHLH) family protein |
| *AT1G09795.1 | 4 | 0.0017% | 1 | 0.0002% | ATP phosphoribosyl transferase 2 (ATP-PRT2) |
| AT3G05060.1 | 4 | 0.0017% | 2 | 0.0004% | SAR DNA-binding protein, putative |
| *AT3G46010.1 | 4 | 0.0017% | 1 | 0.0002% | actin-depolymerizing factor 1 (ADF1) |
| *AT1G77610.1 | 4 | 0.0017% | 1 | 0.0002% | glucose-6-phosphate/phosphate translocator-related |
| *AT4G26140.2 | 4 | 0.0017% | 1 | 0.0002% | beta-galactosidase, putative / lactase, putative |
| AT2G44610.1 | 4 | 0.0017% | 2 | 0.0004% | Ras-related GTP-binding protein, putative |
| AT5G47570.1 | 4 | 0.0017% | 2 | 0.0004% | expressed protein |
| AT1G45170.1 | 4 | 0.0017% | 2 | 0.0004% | expressed protein |
| *AT5G23900.1 | 4 | 0.0017% | 1 | 0.0002% | 60S ribosomal protein L13 (RPL13D) |
| *AT3G55500.1 | 4 | 0.0017% | 1 | 0.0002% | expansin, putative (EXP16) |
| *AT4G25550.1 | 4 | 0.0017% | 1 | 0.0002% | expressed protein |
| *AT1G65650.1 | 4 | 0.0017% | 1 | 0.0002% | ubiquitin carboxyl-terminal hydrolase family 1 protein |
| *AT5G54650.2 | 4 | 0.0017% | 1 | 0.0002% | formin homology 2 domain-containing protein / FH2 domain-containing protein |
| AT5G05620.1 | 4 | 0.0017% | 2 | 0.0004% | tubulin gamma-2 chain / gamma-2 tubulin (TUBG2) |
| AT3G12620.2 | 4 | 0.0017% | 2 | 0.0004% | similar to serine/threonine protein phosphatase 2C (PP2C6) |
| AT5G04410.1 | 4 | 0.0017% | 2 | 0.0004% | no apical meristem (NAM) family protein |
| *AT3G13450.1 | 4 | 0.0017% | 1 | 0.0002% | 2-oxoisovalerate dehydrogenase / 3-methyl-2-oxobutanoate dehydrogenase |
| AT3G54960.1 | 4 | 0.0017% | 2 | 0.0004% | protein disulfide isomerase-like (PDIL) protein |
| *AT3G19820.2 | 4 | 0.0017% | 1 | 0.0002% | cell elongation protein / DWARF1 / DIMINUTO (DIM) |
| AT2G26300.1 | 4 | 0.0017% | 2 | 0.0004% | guanine nucleotide binding protein (G-protein) alpha-1 subunit / GP-alpha-1 (GPA1) |
| *AT1G10730.1 | 4 | 0.0017% | 1 | 0.0002% | clathrin adaptor complexes medium subunit family protein |
| AT2G22480.1 | 4 | 0.0017% | 2 | 0.0004% | phosphofructokinase family protein |
| ATCG00820.1 | 4 | 0.0017% | 2 | 0.0004% | Encodes a 6.8-kDa protein of the small ribosomal subunit |
| AT5G64640.1 | 4 | 0.0017% | 2 | 0.0004% | pectinesterase family protein |
| AT2G32830.1 | 4 | 0.0017% | 2 | 0.0004% | inorganic phosphate transporter (PHT5) |
| AT4G35310.1 | 4 | 0.0017% | 1 | 0.0002% | calcium-dependent protein kinase, putative / CDPK, putative |
| AT5G57230.1 | 3 | 0.0013% | 1 | 0.0002% | expressed protein |
| AT1G56120.1 | 3 | 0.0013% | 1 | 0.0002% | leucine-rich repeat family protein / protein kinase family protein |
| ATMG00580.1 | 3 | 0.0013% | 1 | 0.0002% | NADH dehydrogenase subunit 4 |
| AT1G59870.1 | 3 | 0.0013% | 2 | 0.0004% | ABC transporter family protein |
| AT4G27745.1 | 3 | 0.0013% | 2 | 0.0004% | similar to yippee family protein |
| AT1G72090.1 | 3 | 0.0013% | 2 | 0.0004% | radical SAM domain-containing protein / TRAM domain-containing protein |
| AT5G50950.2 | 3 | 0.0013% | 1 | 0.0002% | fumarate hydratase, putative / fumarase, putative |
| AT3G43700.1 | 3 | 0.0013% | 1 | 0.0002% | speckle-type POZ protein-related, contains Pfam PF00651 : BTB/POZ domain |
| AT2G47180.1 | 3 | 0.0013% | 2 | 0.0004% | galactinol synthase, putative |
| AT4G18170.1 | 3 | 0.0013% | 1 | 0.0002% | WRKY family transcription factor |
| AT4G26780.1 | 3 | 0.0013% | 1 | 0.0002% | co-chaperone grpE family protein |
| AT1G01720.1 | 3 | 0.0013% | 2 | 0.0004% | no apical meristem (NAM) family protein |
| AT5G20680.1 | 3 | 0.0013% | 2 | 0.0004% | expressed protein, predicted proteins |
| AT4G16144.1 | 3 | 0.0013% | 2 | 0.0004% | expressed protein |
| AT4G16060.1 | 3 | 0.0013% | 2 | 0.0004% | expressed protein |
| AT1G55090.1 | 3 | 0.0013% | 1 | 0.0002% | carbon-nitrogen hydrolase family protein |
| AT1G52300.1 | 3 | 0.0013% | 2 | 0.0004% | 60S ribosomal protein L37 (RPL37B) |
| AT4G25310.1 | 3 | 0.0013% | 1 | 0.0002% | oxidoreductase, 2OG-Fe(II) oxygenase family protein |
| AT5G24760.2 | 3 | 0.0013% | 2 | 0.0004% | alcohol dehydrogenase, putative |
| AT5G13750.3 | 3 | 0.0013% | 1 | 0.0002% | similar to sugar transporter family protein |
| AT1G74320.1 | 3 | 0.0013% | 2 | 0.0004% | choline kinase, putative |
| AT3G11170.1 | 3 | 0.0013% | 2 | 0.0004% | omega-3 fatty acid desaturase, chloroplast (FAD7) (FADD) |
| AT3G01690.1 | 3 | 0.0013% | 1 | 0.0002% | expressed protein |
| AT1G30820.1 | 3 | 0.0013% | 2 | 0.0004% | CTP synthase, putative / UTP--ammonia ligase, putative |
| AT5G58390.1 | 3 | 0.0013% | 2 | 0.0004% | peroxidase, putative |
| AT5G22790.1 | 3 | 0.0013% | 1 | 0.0002% | expressed protein |
| AT5G16240.1 | 3 | 0.0013% | 2 | 0.0004% | acyl-(acyl-carrier-protein) desaturase, putative / stearoyl-ACP desaturase, putative |
| AT2G04700.1 | 3 | 0.0013% | 1 | 0.0002% | ferredoxin thioredoxin reductase catalytic beta chain family protein |
| AT3G10530.1 | 3 | 0.0013% | 1 | 0.0002% | transducin family protein / WD-40 repeat family protein |
| AT4G39010.1 | 3 | 0.0013% | 1 | 0.0002% | glycosyl hydrolase family 9 protein, endo-1,4-beta-glucanase precursor - Fragariax ananassa |
| AT5G49690.1 | 3 | 0.0013% | 1 | 0.0002% | UDP-glucoronosyl/UDP-glucosyl transferase family protein |
| AT5G47310.1 | 3 | 0.0013% | 2 | 0.0004% | expressed protein |
| AT1G74670.1 | 3 | 0.0013% | 2 | 0.0004% | gibberellin-responsive protein, putative |
| AT4G12460.1 | 3 | 0.0013% | 2 | 0.0004% | oxysterol-binding family protein |
| AT5G05340.1 | 3 | 0.0013% | 1 | 0.0002% | peroxidase, putative |
| AT4G14100.1 | 3 | 0.0013% | 1 | 0.0002% | expressed protein |
| ATCG01090.1 | 3 | 0.0013% | 1 | 0.0002% | Encodes subunit of the chloroplast NAD(P)H dehydrogenase complex |
| AT2G37190.1 | 3 | 0.0013% | 1 | 0.0002% | 60S ribosomal protein L12 (RPL12A) |
| AT1G27400.1 | 3 | 0.0013% | 2 | 0.0004% | 60S ribosomal protein L17 (RPL17A) |
| AT2G17890.1 | 3 | 0.0013% | 1 | 0.0002% | calcium-dependent protein kinase family protein / CDPK family protein |
| AT2G31790.1 | 3 | 0.0013% | 1 | 0.0002% | UDP-glucoronosyl/UDP-glucosyl transferase family protein |
| AT5G50000.1 | 3 | 0.0013% | 1 | 0.0002% | protein kinase, putative |
| AT1G65700.2 | 3 | 0.0013% | 1 | 0.0002% | similar to small nuclear ribonucleoprotein, putative / snRNP, putative / Sm protein, putative |
| AT2G26640.1 | 3 | 0.0013% | 2 | 0.0004% | beta-ketoacyl-CoA synthase, putative |
| AT5G64760.2 | 3 | 0.0013% | 2 | 0.0004% | similar to 26S proteasome regulatory subunit, putative (RPN5) |
| AT1G72000.1 | 3 | 0.0013% | 2 | 0.0004% | beta-fructofuranosidase, putative / invertase, putative / saccharase, putative / beta-fructosidase, putative |
| AT2G45530.1 | 3 | 0.0013% | 2 | 0.0004% | zinc finger (C3HC4-type RING finger) family protein |
| AT1G02410.1 | 3 | 0.0013% | 1 | 0.0002% | cytochrome c oxidase assembly protein CtaG / Cox11 family |
| AT3G49430.1 | 3 | 0.0013% | 2 | 0.0004% | pre-mRNA splicing factor, putative |
| AT2G31390.1 | 3 | 0.0013% | 2 | 0.0004% | pfkB-type carbohydrate kinase family protein |
| AT1G78300.1 | 3 | 0.0013% | 2 | 0.0004% | 14-3-3 protein GF14 omega (GRF2) |
| AT1G73660.1 | 3 | 0.0013% | 2 | 0.0004% | protein kinase family protein |
| AT5G43370.1 | 3 | 0.0013% | 1 | 0.0002% | inorganic phosphate transporter (PHT2) |
| AT4G01870.1 | 3 | 0.0013% | 2 | 0.0004% | tolB protein-related |
| AT5G17620.1 | 3 | 0.0013% | 2 | 0.0004% | expressed protein |
| AT2G41860.2 | 3 | 0.0013% | 2 | 0.0004% | calcium-dependent protein kinase, putative / CDPK, putative |
| AT4G14540.1 | 3 | 0.0013% | 1 | 0.0002% | CCAAT-box binding transcription factor subunit B (NF-YB) (HAP3 ) (AHAP3) family |
| AT3G63490.2 | 3 | 0.0013% | 1 | 0.0002% | ribosomal protein L1 family protein, ribosomal protein L1, S.oleracea |
| AT5G11880.1 | 3 | 0.0013% | 1 | 0.0002% | diaminopimelate decarboxylase, putative / DAP carboxylase, putative |
| AT3G49390.2 | 3 | 0.0013% | 2 | 0.0004% | similar to RNA-binding protein, putative |
| AT2G33540.1 | 3 | 0.0013% | 1 | 0.0002% | CTD phosphatase-like protein 3 (CPL3) |
| ATCG00750.1 | 3 | 0.0013% | 1 | 0.0002% | 30S chloroplast ribosomal protein S11 |
| AT1G74590.1 | 3 | 0.0013% | 1 | 0.0002% | glutathione S-transferase, putative |
| AT1G31480.1 | 3 | 0.0013% | 2 | 0.0004% | shoot gravitropism 2 (SGR2) |
| AT1G53000.1 | 3 | 0.0013% | 1 | 0.0002% | cytidylyltransferase family |
| AT1G80400.1 | 3 | 0.0013% | 2 | 0.0004% | zinc finger (C3HC4-type RING finger) family protein |
| AT5G41315.1 | 3 | 0.0013% | 2 | 0.0004% | basic helix-loop-helix (bHLH) family protein |
| AT5G41260.1 | 3 | 0.0013% | 2 | 0.0004% | protein kinase family protein |
| AT3G59890.1 | 3 | 0.0013% | 1 | 0.0002% | dihydrodipicolinate reductase family protein |
| AT4G33780.1 | 3 | 0.0013% | 1 | 0.0002% | expressed protein |
| AT1G17890.1 | 3 | 0.0013% | 2 | 0.0004% | GDP-4-keto-6-deoxy-D-mannose-3,5-epimerase-4-reductase, putative |
| AT5G63310.1 | 3 | 0.0013% | 1 | 0.0002% | nucleotide diphosphate kinase II, chloroplast (NDPK2) |
| AT5G17010.2 | 3 | 0.0013% | 1 | 0.0002% | sugar transporter family protein |
| AT1G17870.1 | 3 | 0.0013% | 1 | 0.0002% | S2P-like putative metalloprotease |
| AT3G07680.1 | 3 | 0.0013% | 2 | 0.0004% | emp24/gp25L/p24 family protein |
| AT1G09080.1 | 3 | 0.0013% | 1 | 0.0002% | luminal binding protein 3 (BiP-3) (BP3) |
| AT2G41670.1 | 3 | 0.0013% | 2 | 0.0004% | GTP-binding family protein |
| AT5G48880.3 | 3 | 0.0013% | 1 | 0.0002% | similar to acetyl-CoA C-acyltransferase, putative / 3-ketoacyl-CoA thiolase, putative |
| AT5G22880.1 | 3 | 0.0013% | 1 | 0.0002% | histone H2B, putative |
| AT5G23535.1 | 3 | 0.0013% | 2 | 0.0004% | KOW domain-containing protein |
| AT5G08590.1 | 3 | 0.0013% | 2 | 0.0004% | serine/threonine protein kinase (ASK2) |
| AT3G51310.1 | 3 | 0.0013% | 1 | 0.0002% | vacuolar protein sorting-associated protein 35 family protein / VPS35 family protein |
| AT4G21990.1 | 3 | 0.0013% | 2 | 0.0004% | 5'-adenylylsulfate reductase (APR3) / PAPS reductase homolog (PRH26) |
| AT3G61460.1 | 3 | 0.0013% | 2 | 0.0004% | zinc finger (C3HC4-type RING finger) family protein (BRH1) |
| AT5G61410.2 | 3 | 0.0013% | 2 | 0.0004% | ribulose-phosphate 3-epimerase, chloroplast, putative / pentose-5-phosphate 3-epimerase, putative |
| AT5G20020.1 | 3 | 0.0013% | 1 | 0.0002% | Ras-related GTP-binding nuclear protein (RAN-2) |
| AT5G66060.1 | 3 | 0.0013% | 1 | 0.0002% | oxidoreductase, 2OG-Fe(II) oxygenase family protein |
| AT5G45560.1 | 3 | 0.0013% | 1 | 0.0002% | pleckstrin homology (PH) domain-containing protein / lipid-binding START domain-containing protein |
| AT5G13410.1 | 3 | 0.0013% | 2 | 0.0004% | immunophilin / FKBP-type peptidyl-prolyl cis-trans isomerase family protein |
| AT2G31610.1 | 3 | 0.0013% | 2 | 0.0004% | 40S ribosomal protein S3 (RPS3A) |
| AT2G23420.1 | 3 | 0.0013% | 2 | 0.0004% | nicotinate phosphoribosyltransferase family protein / NAPRTase family protein |
| AT1G79470.1 | 3 | 0.0013% | 2 | 0.0004% | inosine-5'-monophosphate dehydrogenase |
| AT5G07470.1 | 3 | 0.0013% | 2 | 0.0004% | peptide methionine sulfoxide reductase (MSR) |
| AT5G14210.1 | 3 | 0.0013% | 2 | 0.0004% | leucine-rich repeat transmembrane protein kinase, putative |
| AT1G64040.1 | 3 | 0.0013% | 2 | 0.0004% | serine/threonine protein phosphatase PP1 isozyme 3 (TOPP3) / phosphoprotein phosphatase 1 |
| AT1G73270.1 | 3 | 0.0013% | 2 | 0.0004% | serine carboxypeptidase S10 family protein |
| AT4G22990.1 | 3 | 0.0013% | 2 | 0.0004% | SPX (SYG1/Pho81/XPR1) domain-containing protein |
| AT4G03205.2 | 3 | 0.0013% | 1 | 0.0002% | similar to coproporphyrinogen III oxidase, putative / coproporphyrinogenase, putative / coprogen oxidase |
| AT3G02460.2 | 3 | 0.0013% | 2 | 0.0004% | plant adhesion molecule, putative |
| AT2G05940.1 | 3 | 0.0013% | 2 | 0.0004% | protein kinase, putative |
| AT3G53030.1 | 3 | 0.0013% | 1 | 0.0002% | protein kinase family protein |
| AT5G13280.1 | 3 | 0.0013% | 1 | 0.0002% | aspartate kinase |
| AT5G06050.1 | 3 | 0.0013% | 2 | 0.0004% | dehydration-responsive protein-related |
| AT1G11180.1 | 3 | 0.0013% | 2 | 0.0004% | secretory carrier membrane protein (SCAMP) family protein |
| AT3G62700.1 | 3 | 0.0013% | 2 | 0.0004% | glutathione-conjugate transporter, putative |
| AT2G46210.1 | 3 | 0.0013% | 1 | 0.0002% | delta-8 sphingolipid desaturase, putative |
