# Supplementary material for: Comparison of the transcriptomes of American chestnut (Castanea dentata) and Chinese chestnut (Castanea mollissima) in response to the chestnut blight infection
Source: BMC Plant Biol. 2009 May 9;9:51. doi: 10.1186/1471-2229-9-51 (PMC2688492; doi:10.1186/1471-2229-9-51)
Supplement: Additional File 3 — Disease and Defense Response Genes More Highly Expressed in Infected Tissues of Chinese Chestnut (CC) than in American Chestnut (AC). [file 1471-2229-9-51-S3.doc]

**Additional file 3. Disease and Defense** **Response Genes More Highly Expressed in Infected Tissues of Chinese Chestnut (CC) than in American Chestnut (AC). (*)** indicate significant differential expression at 95% confidence level.

| **Arabidopsis Accession #** | **# CC Canker reads** | **% CC Canker transcriptome** | **# AC Canker reads** | **% AC Canker transcriptome** | **Description (Annotation in Arabidopsis)** |
| --- | --- | --- | --- | --- | --- |
| AT5G36880.2 | 12 | 0.51% | 2 | 0.15% | AMP binding / acetate-CoA ligase/ catalytic |
| *AT5G18650.1 | 12 | 0.51% | 0 | 0.00% | zinc finger (C3HC4-type RING finger) family protein |
| *AT4G37640.1 | 11 | 0.47% | 0 | 0.00% | calcium-transporting ATPase 2 |
| ATCG00190.1 | 10 | 0.42% | 4 | 0.31% | Chloroplast DNA-dependent RNA polymerase B subunit |
| *AT3G42050.1 | 9 | 0.38% | 0 | 0.00% | vacuolar ATP synthase subunit H family protein |
| *AT5G07390.1 | 8 | 0.34% | 0 | 0.00% | respiratory burst oxidase protein A (RbohA) / NADPH oxidase |
| AT1G63770.1 | 7 | 0.30% | 2 | 0.15% | peptidase M1 family protein |
| ATCG01010.1 | 7 | 0.30% | 2 | 0.15% | Chloroplast encoded NADH dehydrogenase unit |
| AT2G20650.2 | 7 | 0.30% | 0 | 0.00% | zinc finger (C3HC4-type RING finger) family protein |
| AT5G14450.1 | 7 | 0.30% | 0 | 0.00% | GDSL-motif lipase/hydrolase family protein |
| AT5G58860.1 | 7 | 0.30% | 0 | 0.00% | cytochrome P450 86A1 (CYP86) (CYP86A1) |
| AT2G43790.1 | 7 | 0.30% | 0 | 0.00% | mitogen-activated protein kinase |
| AT2G01060.1 | 7 | 0.30% | 0 | 0.00% | myb family transcription factor |
| AT3G01420.1 | 7 | 0.30% | 0 | 0.00% | pathogen-responsive alpha-dioxygenase |
| AT5G01220.1 | 6 | 0.25% | 3 | 0.23% | DAG sulfoquinovosyltransferase |
| ATCG00180.1 | 6 | 0.25% | 2 | 0.15% | RNA polymerase beta' subunit-1 |
| AT1G14810.1 | 6 | 0.25% | 1 | 0.08% | semialdehyde dehydrogenase family protein |
| AT1G63660.2 | 6 | 0.25% | 1 | 0.08% | GMP synthase (glutamine-hydrolyzing) |
| AT3G08590.2 | 6 | 0.25% | 1 | 0.08% | 2,3-biphosphoglycerate-independent phosphoglycerate mutase |
| AT3G60750.1 | 6 | 0.25% | 1 | 0.08% | transketolase, putative |
| AT1G53510.1 | 6 | 0.25% | 0 | 0.00% | similar to mitogen-activated protein kinase |
| AT5G60020.1 | 6 | 0.25% | 0 | 0.00% | laccase |
| ATCG00120.1 | 5 | 0.21% | 3 | 0.23% | Encodes the ATPase alpha subunit |
| AT2G26080.1 | 5 | 0.21% | 2 | 0.15% | glycine dehydrogenase (decarboxylating) |
| AT4G33010.1 | 5 | 0.21% | 2 | 0.15% | glycine dehydrogenase (decarboxylating) |
| AT5G10330.2 | 5 | 0.21% | 2 | 0.15% | EMB2196 (EMBRYO DEFECTIVE 2196) |
| AT5G42740.1 | 5 | 0.21% | 2 | 0.15% | glucose-6-phosphate isomerase |
| AT2G22250.3 | 5 | 0.21% | 1 | 0.08% | AAT/ATAAT/MEE17 (maternal effect embryo arrest 17) |
| AT2G38290.1 | 5 | 0.21% | 1 | 0.08% | ammonium transporter |
| AT3G10050.1 | 5 | 0.21% | 1 | 0.08% | OMR1 (L-O-METHYLTHREONINE RESISTANT 1) |
| AT3G22200.1 | 5 | 0.21% | 1 | 0.08% | POP2 (POLLEN-PISTIL INCOMPATIBILITY 2) |
| AT4G24830.1 | 5 | 0.21% | 1 | 0.08% | arginosuccinate synthase family |
| AT5G08530.1 | 5 | 0.21% | 1 | 0.08% | NADH-ubiquinone oxidoreductase 51 kDa subunit |
| AT5G11770.1 | 5 | 0.21% | 1 | 0.08% | NADH-ubiquinone oxidoreductase 20 kDa subunit |
| AT5G37510.2 | 5 | 0.21% | 1 | 0.08% | EMB1467 (EMBRYO DEFECTIVE 1467); NADH dehydrogenase |
| AT5G63890.2 | 5 | 0.21% | 1 | 0.08% | ATHDH (HISTIDINOL DEHYDROGENASE) |
| AT1G01190.1 | 5 | 0.21% | 0 | 0.00% | cytochrome P450 |
| AT5G04885.1 | 5 | 0.21% | 0 | 0.00% | glycosyl hydrolase family 3 protein |
| AT5G13410.1 | 5 | 0.21% | 0 | 0.00% | immunophilin / FKBP-type peptidyl-prolyl cis-trans isomerase family protein |
| AT1G48090.1 | 5 | 0.21% | 0 | 0.00% | C2 domain-containing protein |
| AT4G29810.2 | 5 | 0.21% | 0 | 0.00% | similar to mitogen-activated protein kinase kinase (MAPKK;MKK1;MEK1) |
| AT1G68050.1 | 5 | 0.21% | 0 | 0.00% | F-box family protein (FKF1) / adagio 3 (ADO3) |
| AT1G73180.1 | 5 | 0.21% | 0 | 0.00% | eukaryotic translation initiation factor-related |
| AT5G07130.1 | 5 | 0.21% | 0 | 0.00% | laccase |
| AT3G47040.1 | 5 | 0.21% | 0 | 0.00% | glycosyl hydrolase family 3 protein |
| AT1G74110.1 | 5 | 0.21% | 0 | 0.00% | cytochrome P450 family protein |
| AT1G17260.1 | 4 | 0.17% | 2 | 0.15% | AHA10 (AUTOINHIBITED H(+)-ATPASE ISOFORM 10) |
| AT5G27470.1 | 4 | 0.17% | 2 | 0.15% | seryl-tRNA synthetase |
| AT1G50200.1 | 4 | 0.17% | 1 | 0.08% | ALATS (ALANYL-TRNA SYNTHETASE) |
| AT2G20420.1 | 4 | 0.17% | 1 | 0.08% | succinyl-CoA ligase (GDP-forming) beta-chain |
| AT2G45290.1 | 4 | 0.17% | 1 | 0.08% | transketolase |
| AT4G13340.1 | 4 | 0.17% | 1 | 0.08% | leucine-rich repeat family protein / extensin family protein |
| AT4G19210.1 | 4 | 0.17% | 1 | 0.08% | ATRLI2 (Arabidopsis thaliana RNase L inhibitor protein 2) |
| AT5G12200.1 | 4 | 0.17% | 1 | 0.08% | dihydropyrimidine amidohydrolase / hydantoinase (PYD2) |
| AT5G13420.1 | 4 | 0.17% | 1 | 0.08% | transaldolase |
| AT5G50850.1 | 4 | 0.17% | 1 | 0.08% | pyruvate dehydrogenase E1 component beta subunit |
| ATCG01040.1 | 4 | 0.17% | 1 | 0.08% | Identical to Cytochrome c biogenesis protein ccsA (ccsA) |
| AT4G22680.1 | 4 | 0.17% | 0 | 0.00% | myb family transcription factor (MYB85) |
| AT5G60580.4 | 4 | 0.17% | 0 | 0.00% | similar to zinc finger (C3HC4-type RING finger) family protein |
| AT3G10640.1 | 4 | 0.17% | 0 | 0.00% | SNF7 family protein |
| AT5G22000.3 | 4 | 0.17% | 0 | 0.00% | zinc finger (C3HC4-type RING finger) family protein |
| AT2G45570.1 | 4 | 0.17% | 0 | 0.00% | cytochrome P450 76C2 |
| AT5G58490.1 | 4 | 0.17% | 0 | 0.00% | cinnamoyl-CoA reductase family |
| AT1G30400.2 | 4 | 0.17% | 0 | 0.00% | ATP-binding cassette transport protein |
| AT2G22360.1 | 4 | 0.17% | 0 | 0.00% | DNAJ heat shock family protein |
| AT1G09770.1 | 4 | 0.17% | 0 | 0.00% | myb family transcription factor |
| AT1G54790.2 | 4 | 0.17% | 0 | 0.00% | GDSL-motif lipase/hydrolase family protein |
| AT2G31570.1 | 4 | 0.17% | 0 | 0.00% | glutathione peroxidase |
| AT1G10210.2 | 4 | 0.17% | 0 | 0.00% | ATMPK1 | |
| AT5G53540.1 | 4 | 0.17% | 0 | 0.00% | MSP1 protein |
| AT5G04410.1 | 4 | 0.17% | 0 | 0.00% | no apical meristem (NAM) family protein |
| AT1G07420.2 | 4 | 0.17% | 0 | 0.00% | sterol 4-alpha-methyl-oxidase 2 (SMO2) |
| AT4G11610.1 | 4 | 0.17% | 0 | 0.00% | C2 domain-containing protein |
| AT3G22860.1 | 4 | 0.17% | 0 | 0.00% | eukaryotic translation initiation factor 3 subunit 8, putative |
| AT4G28610.1 | 4 | 0.17% | 0 | 0.00% | myb family transcription factor |
| AT3G55320.1 | 4 | 0.17% | 0 | 0.00% | ABC transporter family protein |
| AT1G77670.1 | 3 | 0.13% | 2 | 0.15% | aminotransferase class I and II family protein |
| AT3G14790.1 | 3 | 0.13% | 2 | 0.15% | RHM3 (RHAMNOSE BIOSYNTHESIS 3) |
| AT3G14940.1 | 3 | 0.13% | 2 | 0.15% | phosphoenolpyruvate carboxylase |
| AT3G43790.2 | 3 | 0.13% | 2 | 0.15% | ZIFL2 (ZINC INDUCED FACILITATOR-LIKE 2) |
| AT4G35800.1 | 3 | 0.13% | 2 | 0.15% | NRPB1 (RNA POLYMERASE II LARGE SUBUNIT) |
| AT5G60600.2 | 3 | 0.13% | 2 | 0.15% | GcpE (CHLOROPLAST BIOGENESIS 4) |
| AT1G14610.1 | 3 | 0.13% | 1 | 0.08% | TWN2 (TWIN 2) |
| AT1G71750.1 | 3 | 0.13% | 1 | 0.08% | phosphoribosyltransferase family protein |
| AT1G74040.1 | 3 | 0.13% | 1 | 0.08% | IMS1; 2-isopropylmalate synthase |
| AT1G80600.1 | 3 | 0.13% | 1 | 0.08% | acetylornithine aminotransferase |
| AT2G36880.1 | 3 | 0.13% | 1 | 0.08% | methionine adenosyltransferase |
| AT2G38040.2 | 3 | 0.13% | 1 | 0.08% | CAC3 (acetyl co-enzyme A carboxylase carboxyltransferase alpha subunit) |
| AT2G45270.1 | 3 | 0.13% | 1 | 0.08% | glycoprotease M22 family protein |
| AT3G12290.1 | 3 | 0.13% | 1 | 0.08% | tetrahydrofolate dehydrogenase/cyclohydrolase |
| AT3G23940.1 | 3 | 0.13% | 1 | 0.08% | dehydratase family |
| AT3G23990.1 | 3 | 0.13% | 1 | 0.08% | HSP60 (Heat shock protein 60) |
| AT3G48560.1 | 3 | 0.13% | 1 | 0.08% | CSR1 (CHLORSULFURON/IMIDAZOLINONE RESISTANT 1) |
| AT3G57560.1 | 3 | 0.13% | 1 | 0.08% | aspartate/glutamate/uridylate kinase family protein |
| AT3G62150.1 | 3 | 0.13% | 1 | 0.08% | PGP21 (P-GLYCOPROTEIN 21) |
| AT4G00900.1 | 3 | 0.13% | 1 | 0.08% | calcium-transporting ATPase |
| AT4G10320.1 | 3 | 0.13% | 1 | 0.08% | isoleucyl-tRNA synthetase |
| AT4G33680.1 | 3 | 0.13% | 1 | 0.08% | AGD2 (ABERRANT GROWTH AND DEATH 2) |
| AT4G37910.1 | 3 | 0.13% | 1 | 0.08% | MTHSC70-1 (mitochondrial heat shock protein 70-1) |
| AT5G06580.1 | 3 | 0.13% | 1 | 0.08% | FAD linked oxidase family protein |
| AT5G50320.1 | 3 | 0.13% | 1 | 0.08% | ELO3 (ELONGATA 3) |
| AT5G66120.2 | 3 | 0.13% | 1 | 0.08% | 3-dehydroquinate synthase |
| ATCG00480.1 | 3 | 0.13% | 1 | 0.08% | chloroplast-encoded gene for beta subunit of ATP synthase |
| ATCG01050.1 | 3 | 0.13% | 1 | 0.08% | Represents a plastid-encoded subunit of a NAD(P)H dehydrogenase complex |
