# Supplementary material for: Comparison of the transcriptomes of American chestnut (Castanea dentata) and Chinese chestnut (Castanea mollissima) in response to the chestnut blight infection
Source: BMC Plant Biol. 2009 May 9;9:51. doi: 10.1186/1471-2229-9-51 (PMC2688492; doi:10.1186/1471-2229-9-51)
Supplement: Additional File 4 — Disease- and Defense- Response Genes More Highly Expressed in Infected Tissues of American Chestnut (AC) than in Chinese Chestnut (CC). [file 1471-2229-9-51-S4.doc]

**Additional file 4. Disease- and Defense- Response Genes More Highly Expressed in Infected Tissues of American Chestnut (AC) than in Chinese Chestnut (CC). (*)** indicate significant differential expression at 95% confidence level.

| **Arabidopsis Accession #** | **# AC Canker reads** | **% AC Canker transcriptome** | **# CC Canker reads** | **% CC Canker transcriptome** | **Description (Annotation in Arabidopsis)** |
| --- | --- | --- | --- | --- | --- |
| *AT2G36530.1 | 10 | 0.77% | 7 | 0.30% | LOS2 (Low expression of osmotically responsive genes 1) |
| *AT3G06350.1 | 9 | 0.69% | 2 | 0.08% | EMB3004/MEE32 (EMBRYO DEFECTIVE 3004, maternal effect embryo arrest 32) |
| *ATCG01130.1 | 8 | 0.62% | 1 | 0.04% | Identical to Putative membrane protein ycf1 (ycf1-B) |
| *AT5G08790.1 | 7 | 0.54% | 0 | 0.00% | no apical meristem (NAM) family protein |
| *AT1G15520.1 | 6 | 0.46% | 1 | 0.04% | PLEIOTROPIC DRUG RESISTANCE 12 |
| *AT5G17920.1 | 6 | 0.46% | 2 | 0.08% | ATCIMS (COBALAMIN-INDEPENDENT METHIONINE SYNTHASE) |
| *AT5G54160.1 | 6 | 0.46% | 1 | 0.04% | ATOMT1 (O-METHYLTRANSFERASE 1 |
| *ATCG00170.1 | 6 | 0.46% | 2 | 0.08% | RNA polymerase beta' subunit-2 |
| *AT1G01980.1 | 6 | 0.46% | 0 | 0.00% | FAD-binding domain-containing protein |
| *AT4G30600.1 | 5 | 0.39% | 1 | 0.04% | signal recognition particle receptor alpha subunit family protein |
| *AT5G56010.1 | 5 | 0.39% | 1 | 0.04% | HSP81-3 (Heat shock protein 81-3) |
| *AT1G30700.1 | 5 | 0.39% | 0 | 0.00% | FAD-binding domain-containing protein |
| *AT3G17020.1 | 5 | 0.39% | 0 | 0.00% | Universal stress protein (USP) family protein |
| *AT2G19830.1 | 5 | 0.39% | 0 | 0.00% | SNF7 family protein, contains Pfam domain, PF03357 |
| *AT1G72680.1 | 4 | 0.31% | 1 | 0.04% | cinnamyl-alcohol dehydrogenase |
| AT4G01850.1 | 4 | 0.31% | 3 | 0.13% | MAT2/SAM-2 (S-adenosylmethionine synthetase 2); methionine adenosyltransferase |
| AT5G48100.1 | 4 | 0.31% | 2 | 0.08% | TT10 (TRANSPARENT TESTA 10); laccase |
| *AT5G50920.1 | 4 | 0.31% | 1 | 0.04% | CLPC (HEAT SHOCK PROTEIN 93-V) |
| ATCG00490.1 | 4 | 0.31% | 3 | 0.13% | large subunit of RUBISCO |
| *AT2G41560.1 | 4 | 0.31% | 0 | 0.00% | calcium-transporting ATPase 4 |
| *ATCG00730.1 | 4 | 0.31% | 0 | 0.00% | A chloroplast gene encoding subunit IV of the cytochrome b6/f complex |
| *AT1G64160.1 | 4 | 0.31% | 0 | 0.00% | disease resistance-responsive family protein |
| *AT5G60020.1 | 4 | 0.31% | 0 | 0.00% | laccase, putative / diphenol oxidase |
| AT1G09780.1 | 3 | 0.23% | 1 | 0.04% | 2,3-biphosphoglycerate-independent phosphoglycerate mutase |
| AT1G17840.1 | 3 | 0.23% | 1 | 0.04% | ABC transporter family protein |
| AT1G30700.1 | 3 | 0.23% | 1 | 0.04% | FAD-binding domain-containing protein |
| AT1G53920.1 | 3 | 0.23% | 1 | 0.04% | GLIP5 (GDSL-motif lipase 5) |
| AT2G28000.1 | 3 | 0.23% | 1 | 0.04% | CPN60A (chloroplast / 60 kDa chaperonin alpha subunit) |
| AT2G35940.3 | 3 | 0.23% | 1 | 0.04% | EDA29, BLH1 | BLH1 (BLH1) | chr2:15095911-15099027 REVERSE |
| AT2G41530.1 | 3 | 0.23% | 1 | 0.04% | esterase |
| AT3G02090.2 | 3 | 0.23% | 1 | 0.04% | MPPBETA; metalloendopeptidase |
| AT3G02360.1 | 3 | 0.23% | 1 | 0.04% | 6-phosphogluconate dehydrogenase family protein |
| AT3G55440.1 | 3 | 0.23% | 1 | 0.04% | triose-phosphate isomerase |
| AT4G01320.1 | 3 | 0.23% | 2 | 0.08% | STE24, ATSTE24 |
| AT4G09320.1 | 3 | 0.23% | 1 | 0.04% | NDPK1 (nucleoside diphosphate kinase 1) |
| AT4G30190.1 | 3 | 0.23% | 1 | 0.04% | AHA2 (Arabidopsis H(+)-ATPase 2) |
| AT4G34350.1 | 3 | 0.23% | 2 | 0.08% | CLB6 (CHLOROPLAST BIOGENESIS 6) |
| AT5G17330.1 | 3 | 0.23% | 1 | 0.04% | GAD (Glutamate decarboxylase 1) |
| AT5G20980.1 | 3 | 0.23% | 2 | 0.08% | ATMS3 (METHIONINE SYNTHASE 3) |
| AT5G27600.1 | 3 | 0.23% | 1 | 0.04% | LACS7 (LONG-CHAIN ACYL-COA SYNTHETASE 7) |
| AT5G60390.1 | 3 | 0.23% | 1 | 0.04% | elongation factor 1-alpha |
| *AT1G21410.1 | 3 | 0.23% | 0 | 0.00% | F-box family protein |
| *AT3G52450.1 | 3 | 0.23% | 0 | 0.00% | U-box domain-containing protein |
| *AT4G37970.1 | 3 | 0.23% | 0 | 0.00% | mannitol dehydrogenase |
| *AT1G66540.1 | 3 | 0.23% | 0 | 0.00% | cytochrome P450 |
| *AT3G18370.1 | 3 | 0.23% | 0 | 0.00% | C2 domain-containing protein |
| *AT4G01250.1 | 3 | 0.23% | 0 | 0.00% | WRKY11 family transcription factor |
| *AT5G03030.1 | 3 | 0.23% | 0 | 0.00% | DNAJ heat shock N-terminal domain-containing protein |
| *AT4G11810.1 | 3 | 0.23% | 0 | 0.00% | SPX (SYG1/Pho81/XPR1) domain-containing protein |
| *AT1G16490.1 | 3 | 0.23% | 0 | 0.00% | MYB family transcription factor (MYB58) |
| *AT1G19320.1 | 3 | 0.23% | 0 | 0.00% | pathogenesis-related thaumatin family protein |
| *AT2G26850.1 | 3 | 0.23% | 0 | 0.00% | F-box family protein |
| *AT1G26770.1 | 3 | 0.23% | 0 | 0.00% | expansin, putative (EXP10) |
| *AT2G33600.1 | 3 | 0.23% | 0 | 0.00% | cinnamoyl-CoA reductase family |
